# Supplementary material for: Localizations of Laminin Chains Suggest Their Multifaceted Functions in Mouse Tooth Development
Source: Int J Mol Sci. 2025 Apr 26;26(9):4134. doi: 10.3390/ijms26094134 (PMC12071823; doi:10.3390/ijms26094134)
Supplement: Supplementary file 1 [file ijms-26-04134-s001.zip › ijms-3532117-supplementary.pdf]

Supplemental Materials

Localizations of Laminin Chains Suggest Their Multifaceted Functions in Mouse Tooth Development

Tian Liang[1], Hong Zhang[2], Yuanyuan Hu[1], Mansi Solanki[1], Chuhua Zhang[2], Takako Sasaki[3], Charles E. Smith[2,4], Jan C.-C. Hu[2], James P. Simmer[2].

[1]Department of Orthodontics and Pediatric Dentistry, University of Michigan School of Dentistry, 1011 North University, Ann Arbor, MI 48109, USA.

[2]Department of Biologic and Materials Sciences, University of Michigan School of Dentistry, 1011 North University, Ann Arbor, MI 48109, USA.

[3]Department of Matrix Medicine, Faculty of Medicine, Oita University, 1-1 Idaigaoka, Hasama-Machi, Yufu City, Oita, 879-5593, Japan.

[4]Department of Anatomy & Cell Biology, Faculty of Medicine & Health Sciences, McGill University, 3640 University St., Montreal, QC, H3A 0C7, Canada.

Table S1. Riboprobes used in this study

Target Transcript	Probe name	Catalog number	Accession number	Target region
Col4a1	Mm-Col4a1	412871	NM_009931.2	2496 - 3841
Col7a1	Mm-Col7a1	855151	NM_007738.4	109 - 964
Lama1	Mm-Lama1	494931	NM_008480.2	1025 - 1922
Lama2	Mm-Lama2	424661	NM_008481.2	3264 - 4317
Lama3	Mm-Lama3	494921	NM_010680.1	6312 - 7236
Lama4	Mm-Lama4	494901	NM_010681.4	1067 - 1972
Lama5	Mm-Lama5	494911	NM_001081171.2	1193 - 2091
Lamb1	Mm-Lamb1	517641	NM_008482.2	1263 - 2143
Lamb2	Mm-Lamb2	412891	NM_008483.3	1761 - 2744
Lamb3	Mm-Lamb3	552161	NM_008484.2	1929 - 2859
Lamc1	Mm-Lamc1	517451	NM_010683.2	2768 - 3626
Lamc2	Mm-Lamc2-No-XHs	482601	NM_008485.3	2417 - 4331
Lamc3	Mm-Lamc3	842421	NM_011836.4	567 - 1480
DapB (Neg Ctrl)	DapB (Neg Ctrl)	310043	EF191515	414-862

Note: All riboprobes come from Advanced Cell Diagnostics, Inc.

Table S2. Antibodies used in this study

Antigen	Name	Source
type IV collagen	Rabbit polyclonal antibody to type IV collagen	abcam, ab6586
LAMA1	Rabbit monoclonal [EPR27258-37] antibody to LAMA1	abcam, ab305742
LAMA2	Rat monoclonal (4H8-2) antibody to LAMA2	Sigma, L0063
LAMA3	Rabbit antiserum to LAMA3A IIIa	T. Sasaki, #1110+ [23]
LAMA4	Goat polyclonal antibody to LAMA4	R&D System, AF3837
LAMA5	Rabbit monoclonal [EPR18919] antibody to LAMA5	abcam, ab184330
LAMB1	Rat monoclonal (LT3) antibody to LAMB1	Santa Cruz, sc-33709
LAMB3	Rabbit antiserum to LAMB3 VI/V	T. Sasaki, #1111+ [23]
LAMC1	Rabbit monoclonal [EPR21199] antibody to LAMC1	abcam, ab233389
LAMC2	Rabbit antiserum to LAMC2 LE4-6	T. Sasaki, #1097+ [23]
laminin 332	Rabbit polyclonal antibody to laminin 332	abcam, ab14509
Ki67	Rabbit monoclonal (D3B5) antibody to Ki67	Cell Signaling Tech, 12202
Rabbit IgG	Goat anti-rabbit IgG(H+L) highly adsorbed secondary antibody, Alexa Fluor Plus 594	Invitrogen, A32740
Rat IgG	Donkey anti-Rat IgG (H+L) Highly Cross-Adsorbed Secondary Antibody, Alexa Fluor Plus 555	Invitrogen, A48270
Goat IgG	Donkey anti-Goat IgG (H+L) Highly Cross-Adsorbed Secondary Antibody, Alexa Fluor Plus 555	Invitrogen, A32816
β-actin	Mouse monoclonal [AC-15] to β-actin, conjugated with FITC	abcam, ab6277

Note: [23] Sasaki, T.; Göhring, W.; Mann, K.; Brakebusch, C.; Yamada, Y.; Fässler, R.; Timpl, R. Short arm region of laminin-5 gamma2 chain: structure, mechanism of processing and binding to heparin and proteins. *J Mol Biol* **2001**, *314*, 751-763, doi:10.1006/jmbi.2001.5176.

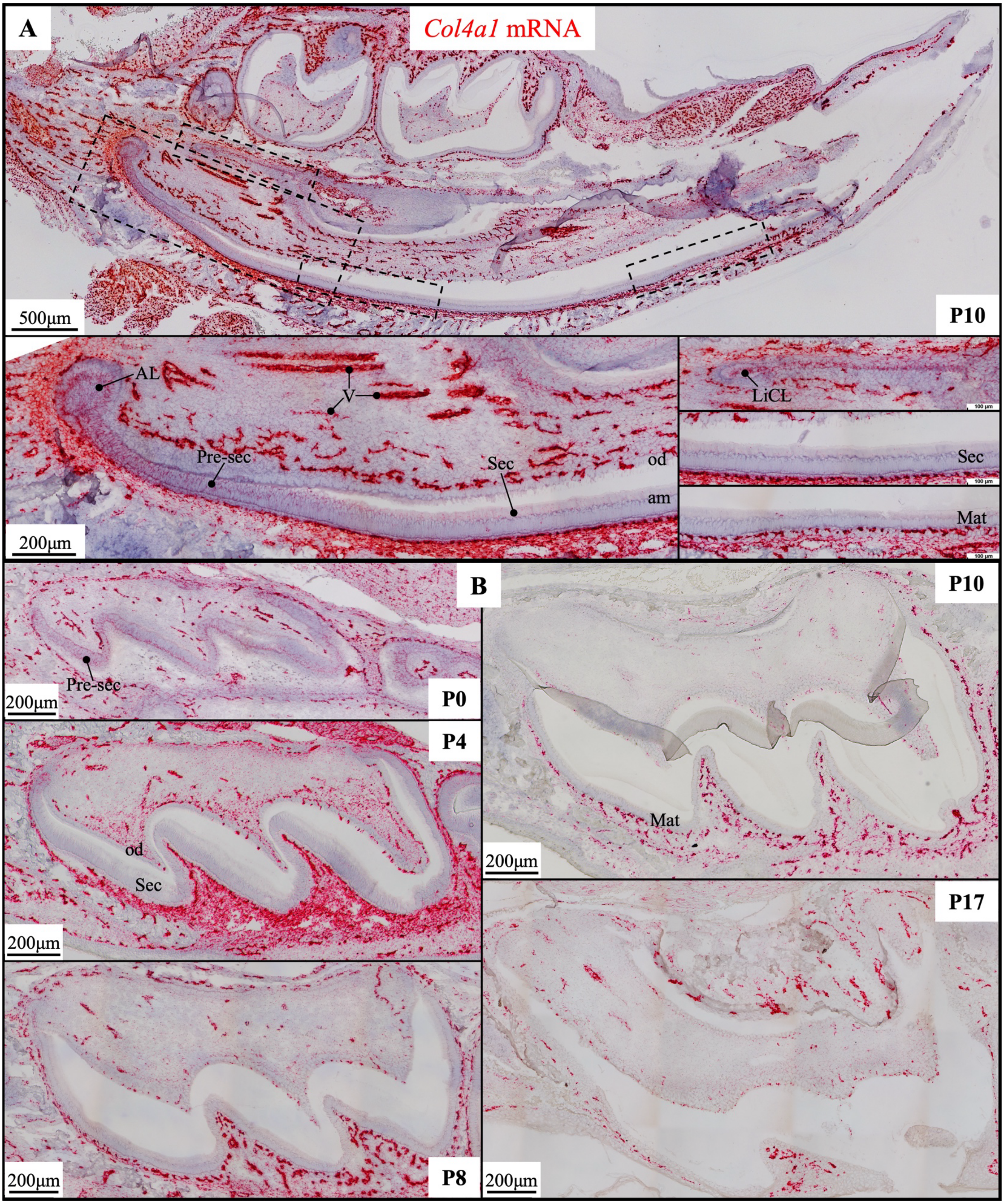

Fig S1. RNAscope in situ hybridization of *Col4a1* in mouse developing teeth. A. 10-day-old (P10) mandibular incisors that contain all ameloblast developmental stages, including the dental epithelial stem cells in the apical loop (AL) that differentiate into pre-secretory (Pre-Sec), secretory (Sec), and maturation (Mat) stage ameloblasts (am). The lingual cervical loop (LiCL) is also shown. **B**. Newborn (P0), 4-day-old (P4), 8-day-old (P8), 10-day-old (P10), and 17-day-old (P17) maxillary 1st molars. Key: od, odontoblasts; V, blood vessels.

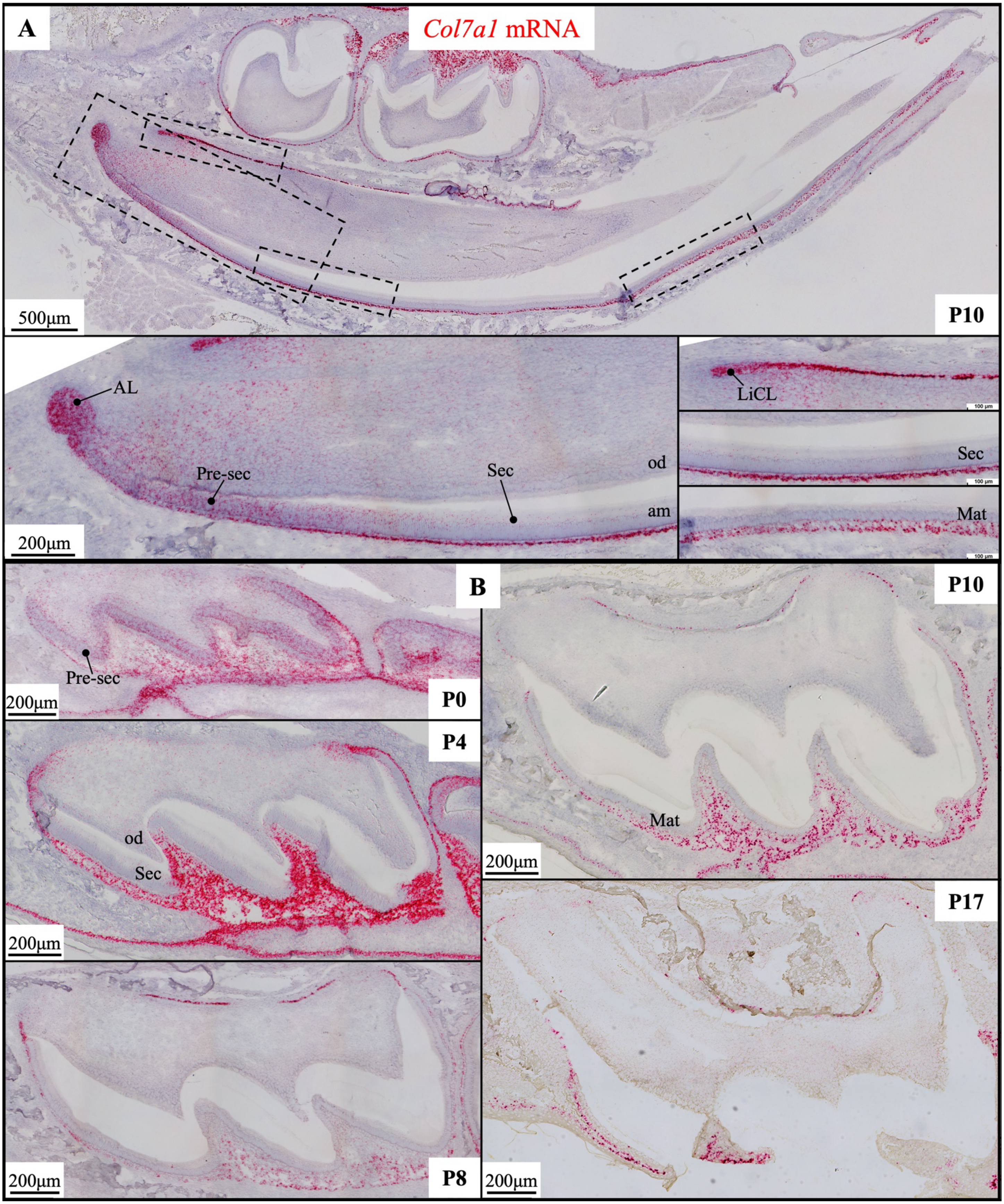

Fig S2. RNAscope in situ hybridization of *Col7a1* in mouse developing teeth. A. 10-day-old (P10) mandibular incisors that contain all ameloblast developmental stages, including the dental epithelial stem cells in the apical loop (AL) that differentiate into pre-secretory (Pre-Sec), secretory (Sec), and maturation (Mat) stage ameloblasts (am). The lingual cervical loop (LiCL) is also shown. **B**. Newborn (P0), 4-day-old (P4), 8-day-old (P8), 10-day-old (P10), and 17-day-old (P17) maxillary 1st molars. Key: od, odontoblasts.

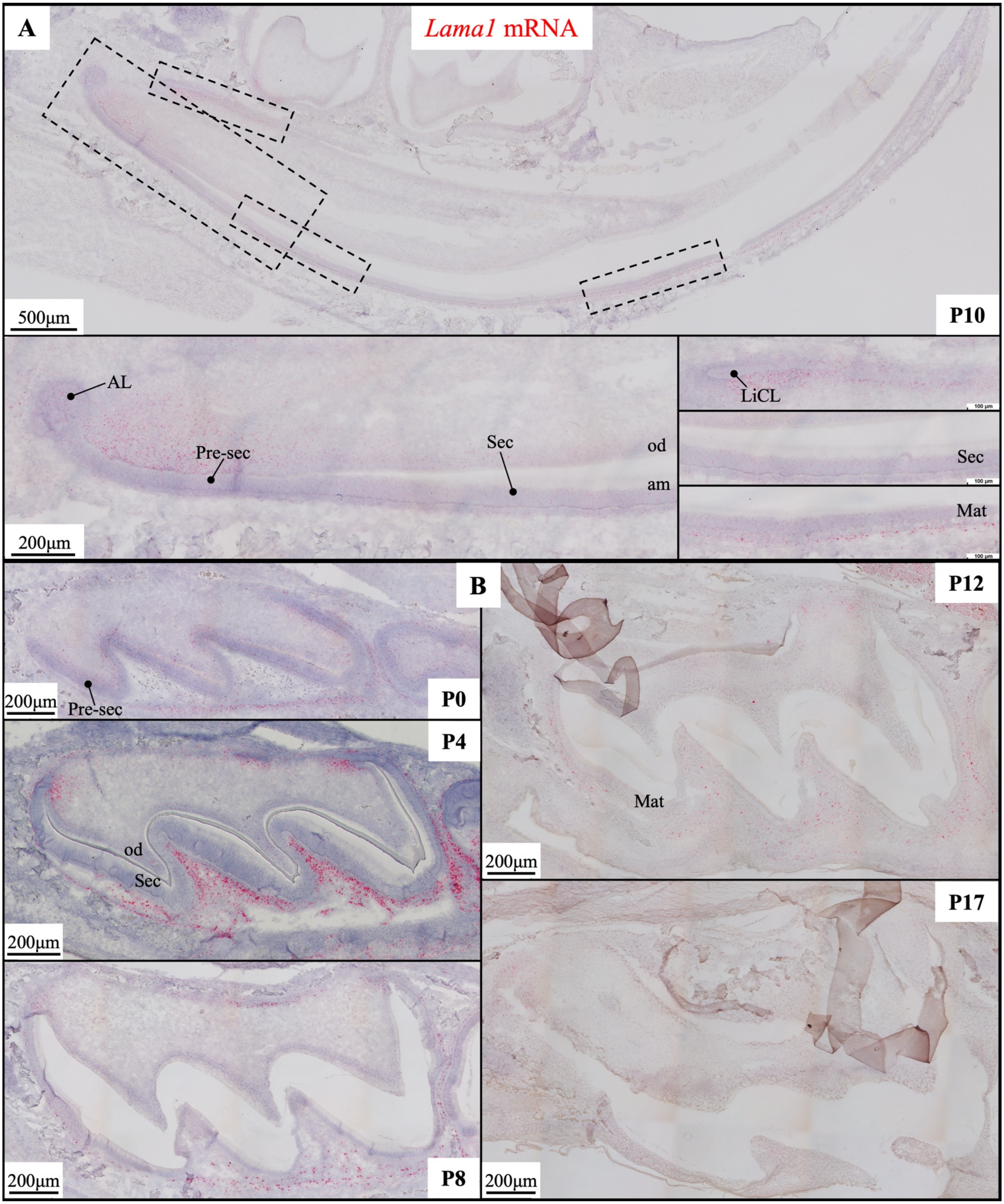

Fig S3. RNAscope in situ hybridization of *Lama1* in mouse developing teeth. A. 10-day-old (P10) mandibular incisors that contain all ameloblast developmental stages, including the dental epithelial stem cells in the apical loop (AL) that differentiate into pre-secretory (Pre-Sec), secretory (Sec), and maturation (Mat) stage ameloblasts (am). The lingual cervical loop (LiCL) is also shown. **B**. Newborn (P0), 4-day-old (P4), 8-day-old (P8), 12-day-old (P12), and 17-day-old (P17) maxillary 1st molars. Key: od, odontoblasts.

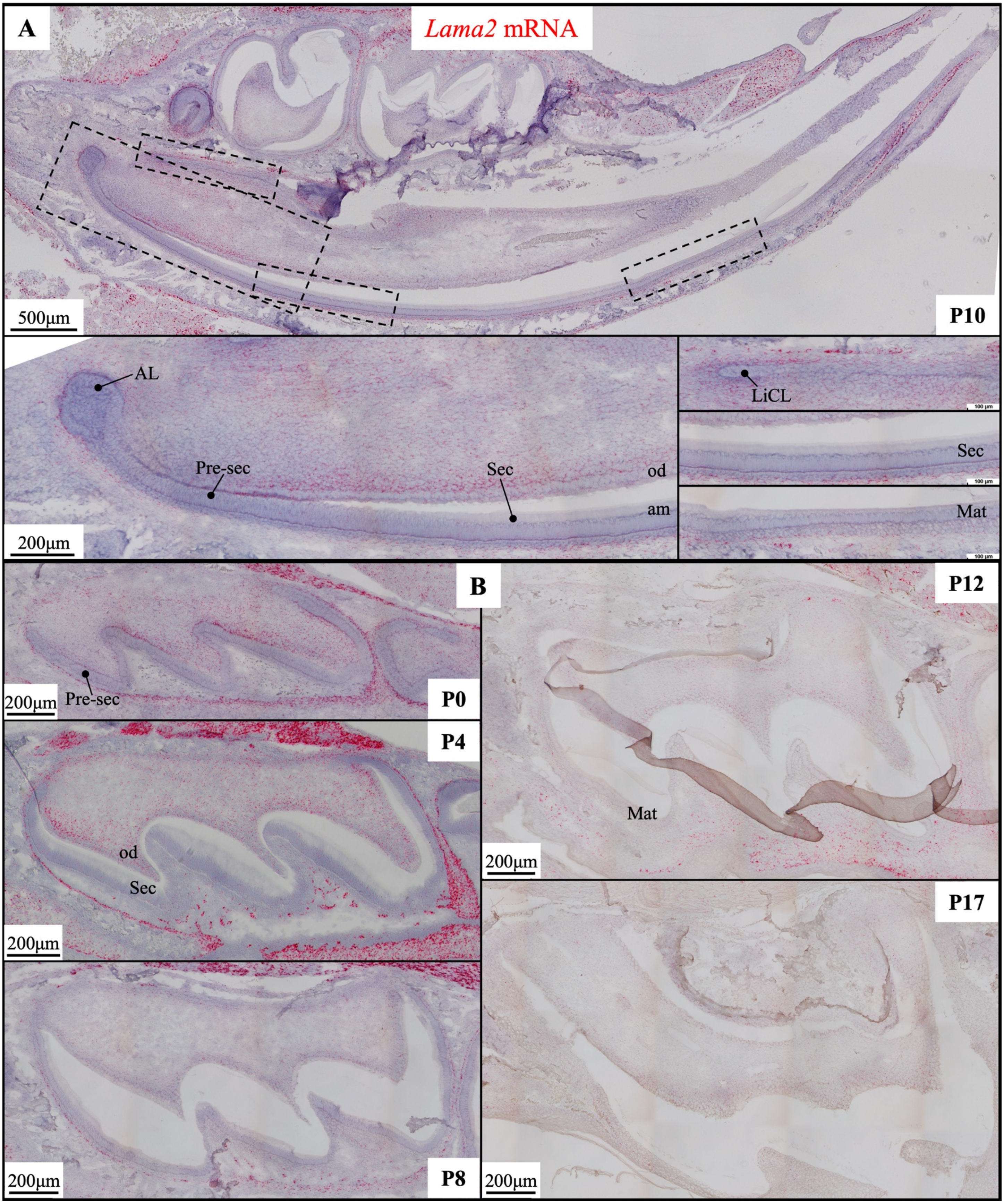

Fig S4. RNAscope in situ hybridization of *Lama2* in mouse developing teeth. A. 10-day-old (P10) mandibular incisors that contain all ameloblast developmental stages, including the dental epithelial stem cells in the apical loop (AL) that differentiate into pre-secretory (Pre-Sec), secretory (Sec), and maturation (Mat) stage ameloblasts (am). The lingual cervical loop (LiCL) is also shown. **B**. Newborn (P0), 4-day-old (P4), 8-day-old (P8), 12-day-old (P12), and 17-day-old (P17) maxillary 1st molars. Key: od, odontoblasts.

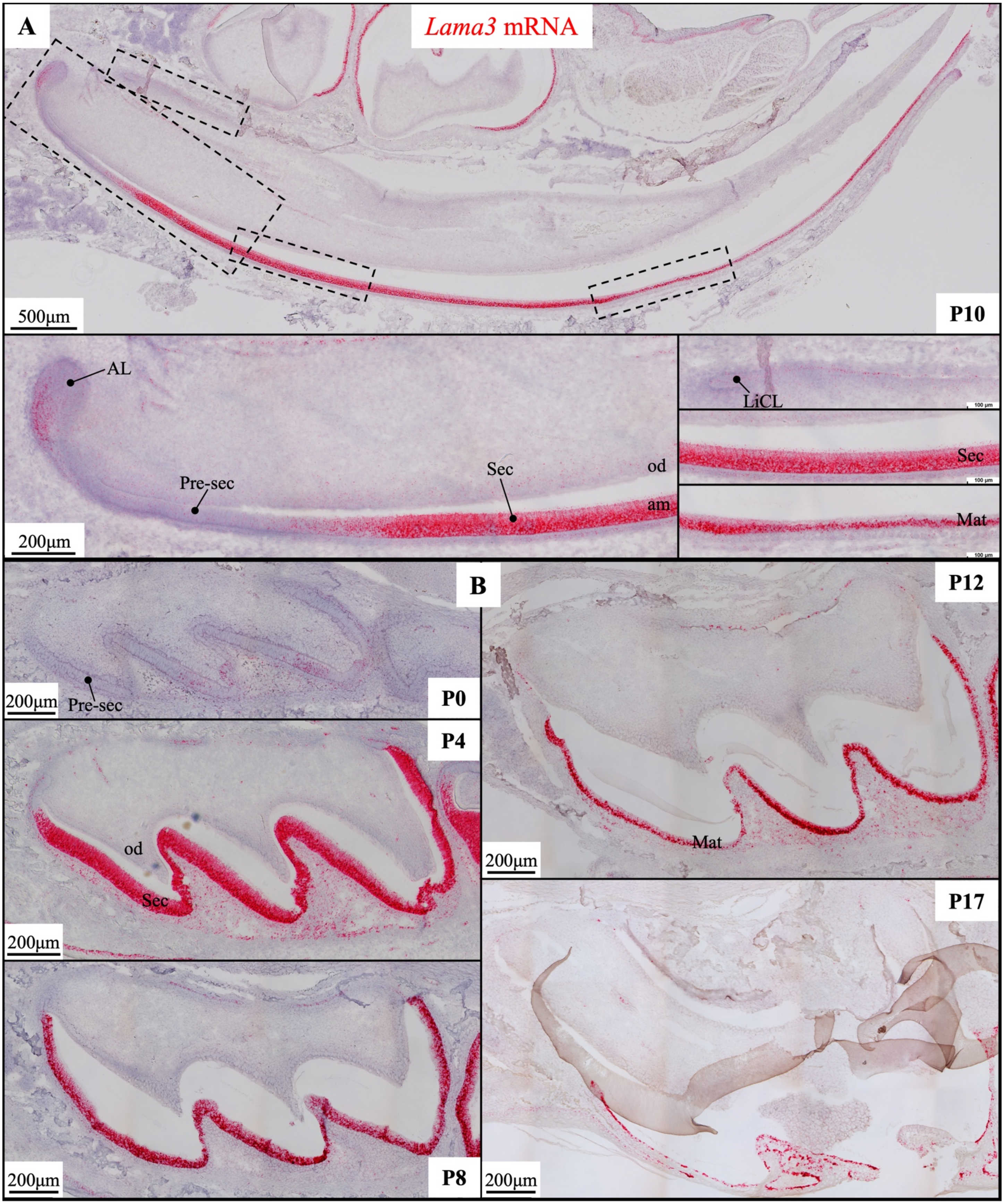

Fig S5. RNAscope in situ hybridization of *Lama3* in mouse developing teeth. A. 10-day-old (P10) mandibular incisors that contain all ameloblast developmental stages, including the dental epithelial stem cells in the apical loop (AL) that differentiate into pre-secretory (Pre-Sec), secretory (Sec), and maturation (Mat) stage ameloblasts (am). The lingual cervical loop (LiCL) is also shown. **B**. Newborn (P0), 4-day-old (P4), 8-day-old (P8), 12-day-old (P12), and 17-day-old (P17) maxillary 1st molars. Key: od, odontoblasts.

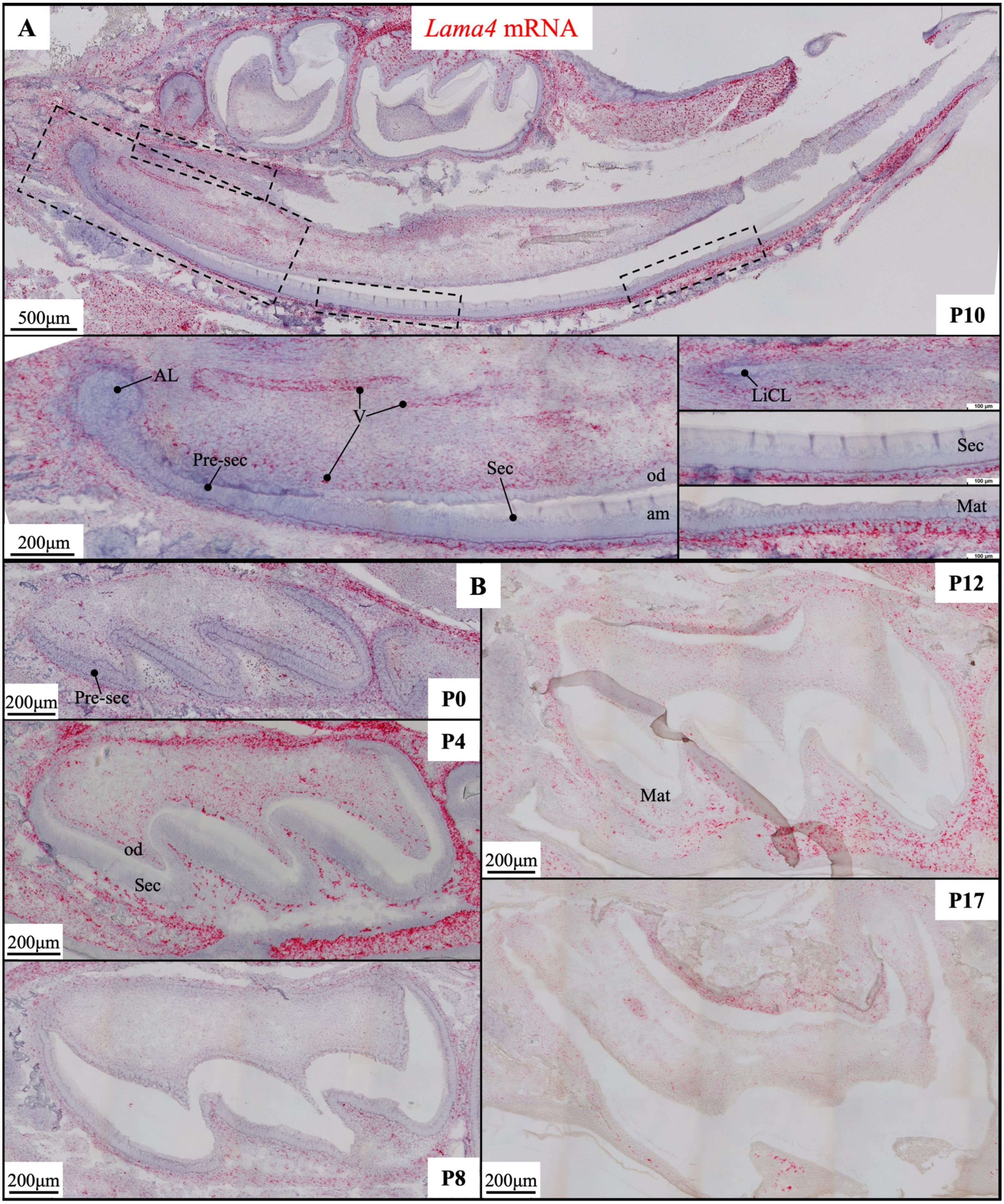

Fig S6. RNAscope in situ hybridization of *Lama4* in mouse developing teeth. A. 10-day-old (P10) mandibular incisors that contain all ameloblast developmental stages, including the dental epithelial stem cells in the apical loop (AL) that differentiate into pre-secretory (Pre-Sec), secretory (Sec), and maturation (Mat) stage ameloblasts (am). The lingual cervical loop (LiCL) is also shown. **B**. Newborn (P0), 4-day-old (P4), 8-day-old (P8), 12-day-old (P12), and 17-day-old (P17) maxillary 1st molars. Key: od, odontoblasts; V, blood vessels.

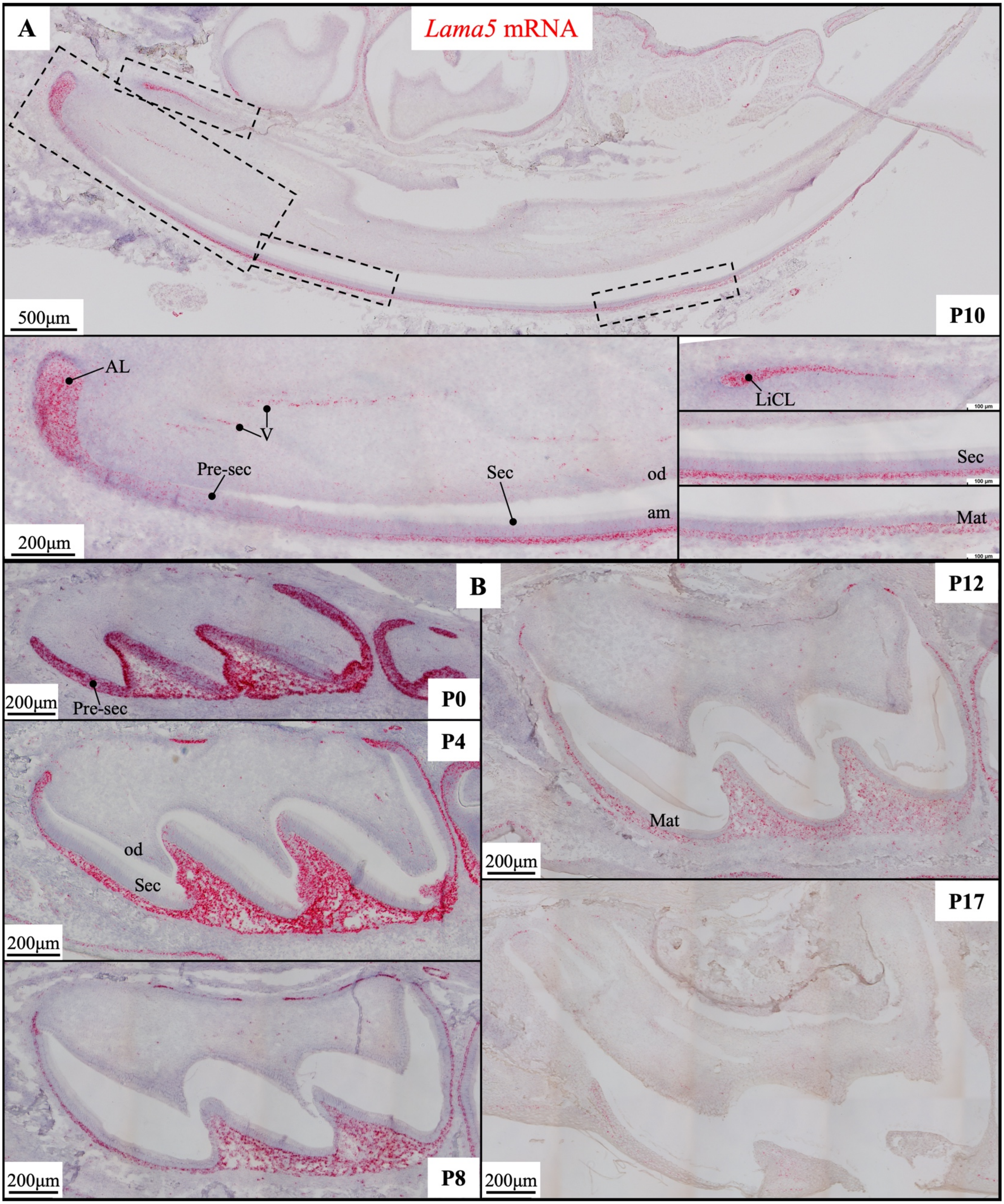

Fig S7. RNAscope in situ hybridization of *Lama5* in mouse developing teeth. A. 10-day-old (P10) mandibular incisors that contain all ameloblast developmental stages, including the dental epithelial stem cells in the apical loop (AL) that differentiate into pre-secretory (Pre-Sec), secretory (Sec), and maturation (Mat) stage ameloblasts (am). The lingual cervical loop (LiCL) is also shown. **B**. Newborn (P0), 4-day-old (P4), 8-day-old (P8), 12-day-old (P12), and 17-day-old (P17) maxillary 1st molars. Key: od, odontoblasts; V, blood vessels.

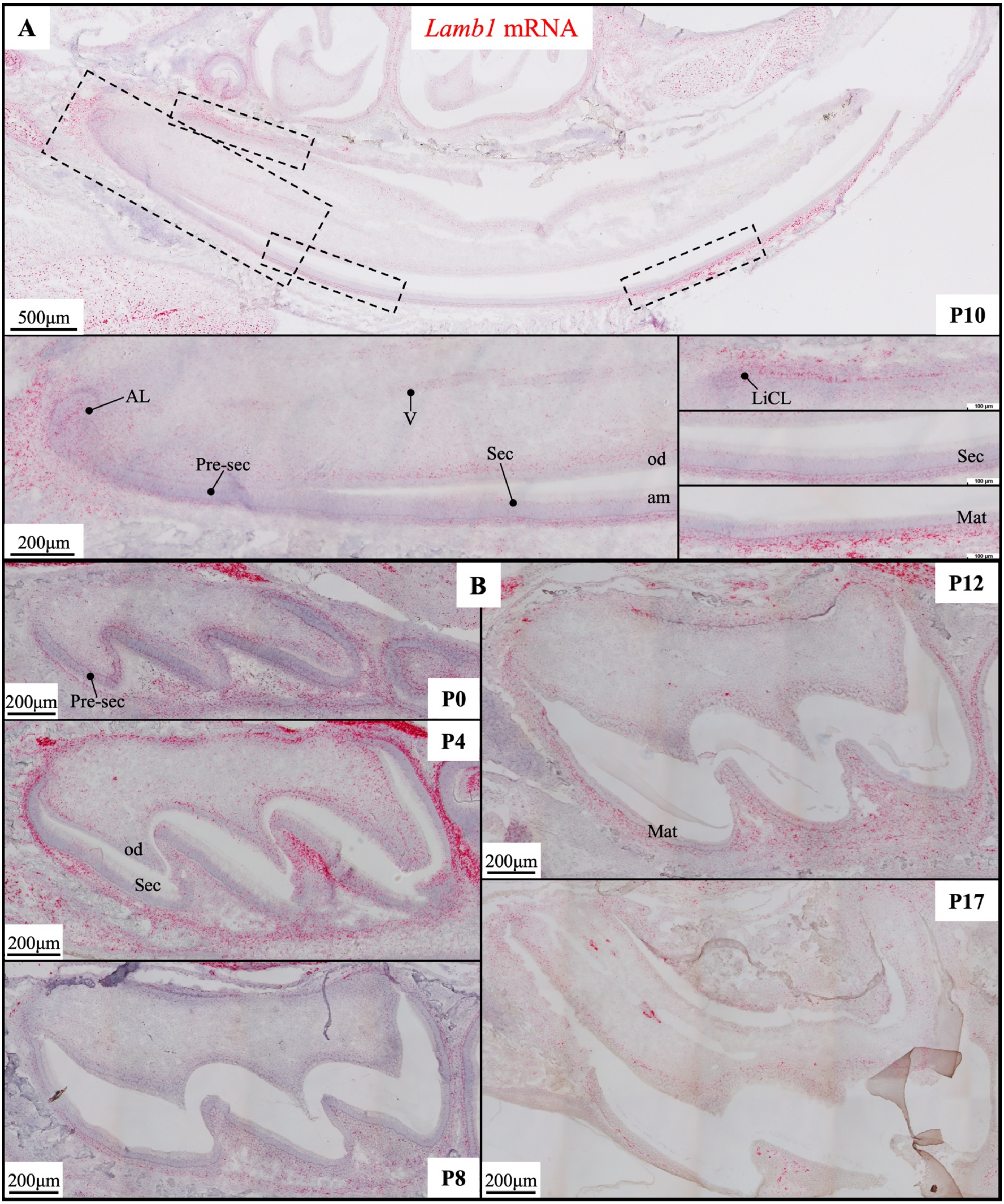

Fig S8. RNAscope in situ hybridization of *Lamb1* in mouse developing teeth. A. 10-day-old (P10) mandibular incisors that contain all ameloblast developmental stages, including the dental epithelial stem cells in the apical loop (AL) that differentiate into pre-secretory (Pre-Sec), secretory (Sec), and maturation (Mat) stage ameloblasts (am). The lingual cervical loop (LiCL) is also shown. **B**. Newborn (P0), 4-day-old (P4), 8-day-old (P8), 12-day-old (P12), and 17-day-old (P17) maxillary 1st molars. Key: od, odontoblasts; V, blood vessels.

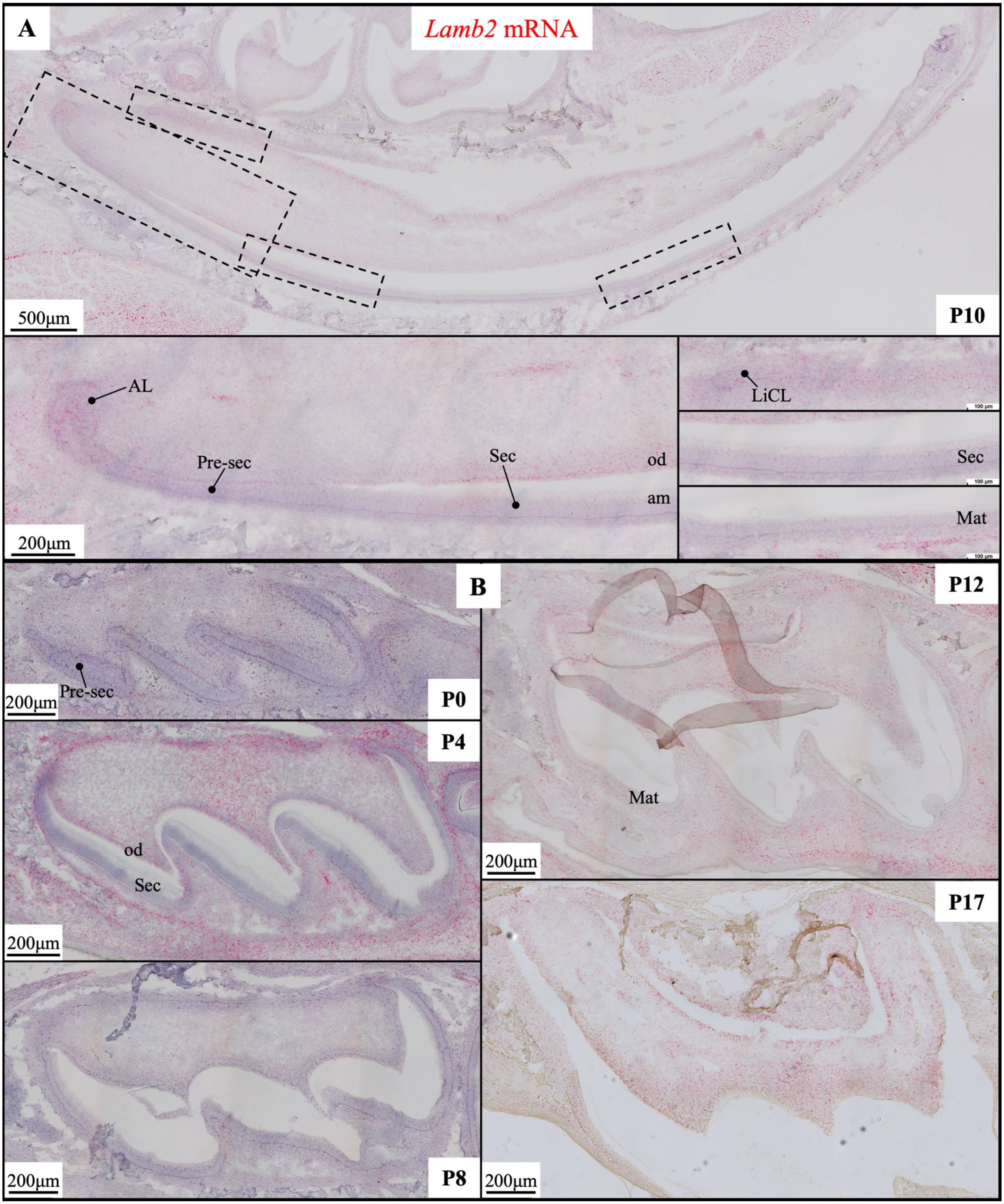

Fig S9. RNAscope in situ hybridization of *Lamb2* in mouse developing teeth. A. 10-day-old (P10) mandibular incisors that contain all ameloblast developmental stages, including the dental epithelial stem cells in the apical loop (AL) that differentiate into pre-secretory (Pre-Sec), secretory (Sec), and maturation (Mat) stage ameloblasts (am). The lingual cervical loop (LiCL) is also shown. **B**. Newborn (P0), 4-day-old (P4), 8-day-old (P8), 12-day-old (P12), and 17-day-old (P17) maxillary 1st molars. Key: od, odontoblasts.

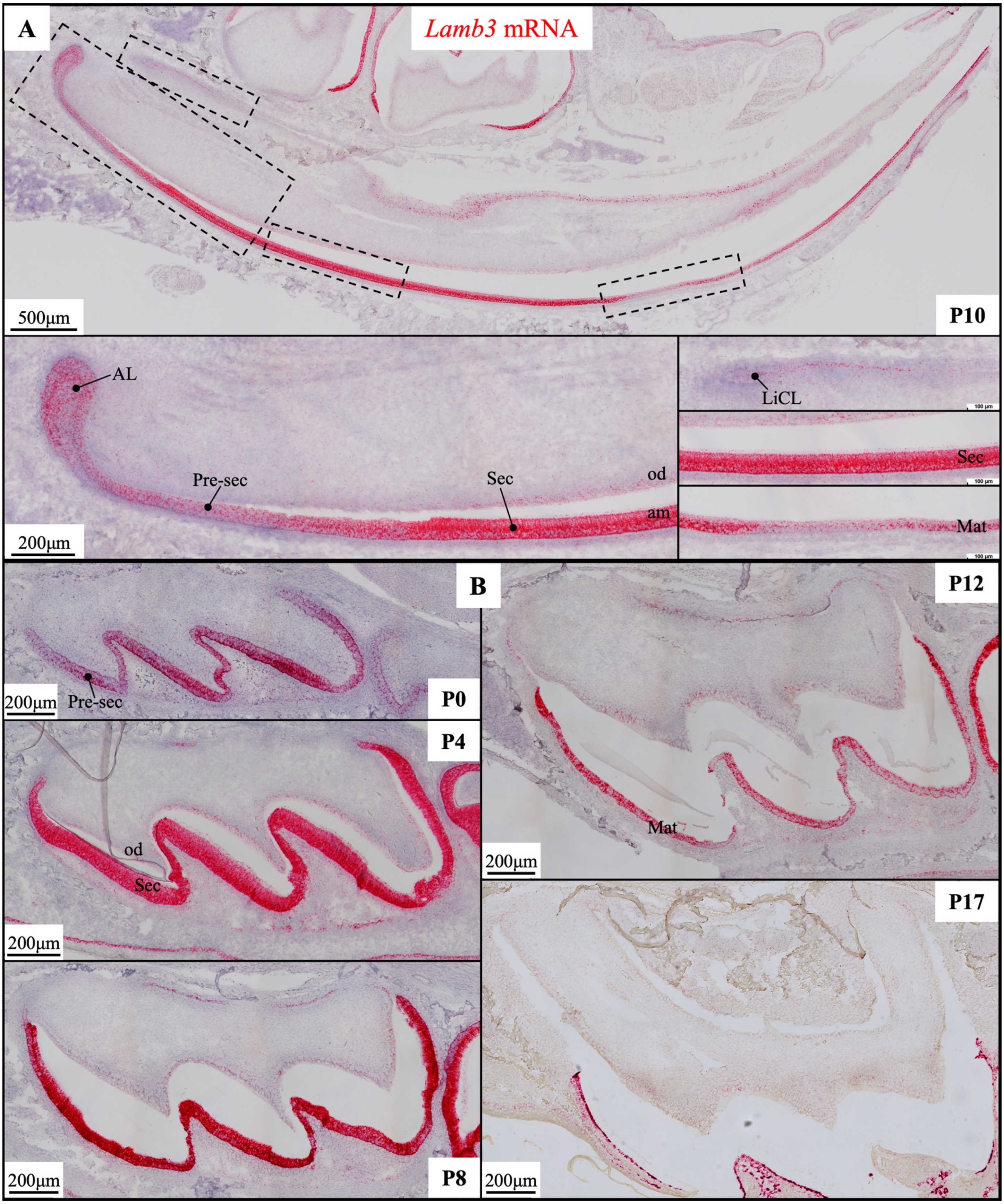

Fig S10. RNAscope in situ hybridization of *Lamb3* in mouse developing teeth. A. 10-day-old (P10) mandibular incisors that contain all ameloblast developmental stages, including the dental epithelial stem cells in the apical loop (AL) that differentiate into pre-secretory (Pre-Sec), secretory (Sec), and maturation (Mat) stage ameloblasts (am). The lingual cervical loop (LiCL) is also shown. **B**. Newborn (P0), 4-day-old (P4), 8-day-old (P8), 12-day-old (P12), and 17-day-old (P17) maxillary 1st molars. Key: od, odontoblasts.

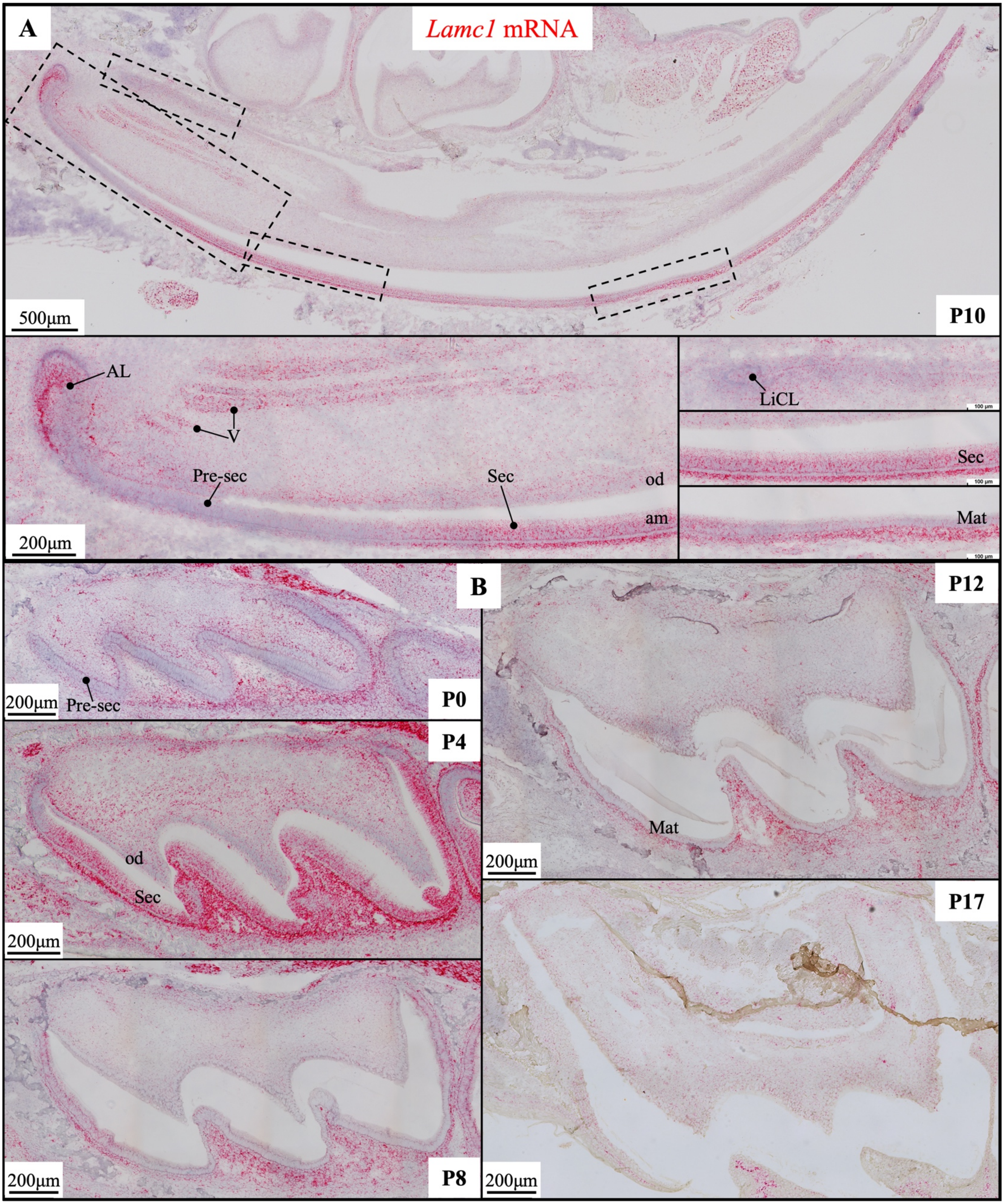

Fig S11. RNAscope in situ hybridization of *Lamc1* in mouse developing teeth. A. 10-day-old (P10) mandibular incisors that contain all ameloblast developmental stages, including the dental epithelial stem cells in the apical loop (AL) that differentiate into pre-secretory (Pre-Sec), secretory (Sec), and maturation (Mat) stage ameloblasts (am). The lingual cervical loop (LiCL) is also shown. **B**. Newborn (P0), 4-day-old (P4), 8-day-old (P8), 12-day-old (P12), and 17-day-old (P17) maxillary 1st molars. Key: od, odontoblasts; V, blood vessels.

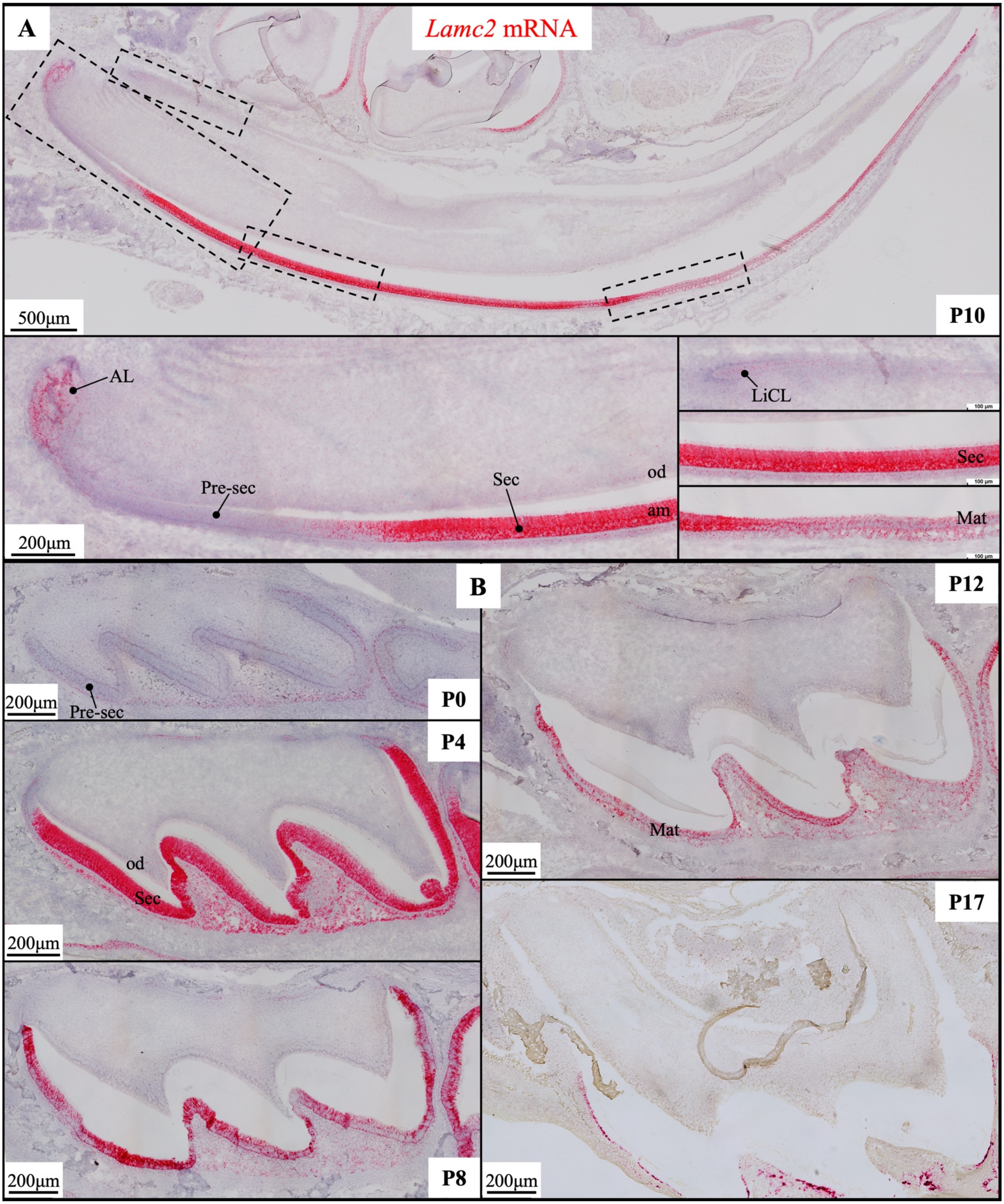

Fig S12. RNAscope in situ hybridization of *Lamc2* in mouse developing teeth. A. 10-day-old (P10) mandibular incisors that contain all ameloblast developmental stages, including the dental epithelial stem cells in the apical loop (AL) that differentiate into pre-secretory (Pre-Sec), secretory (Sec), and maturation (Mat) stage ameloblasts (am). The lingual cervical loop (LiCL) is also shown. **B**. Newborn (P0), 4-day-old (P4), 8-day-old (P8), 12-day-old (P12), and 17-day-old (P17) maxillary 1st molars. Key: od, odontoblasts.

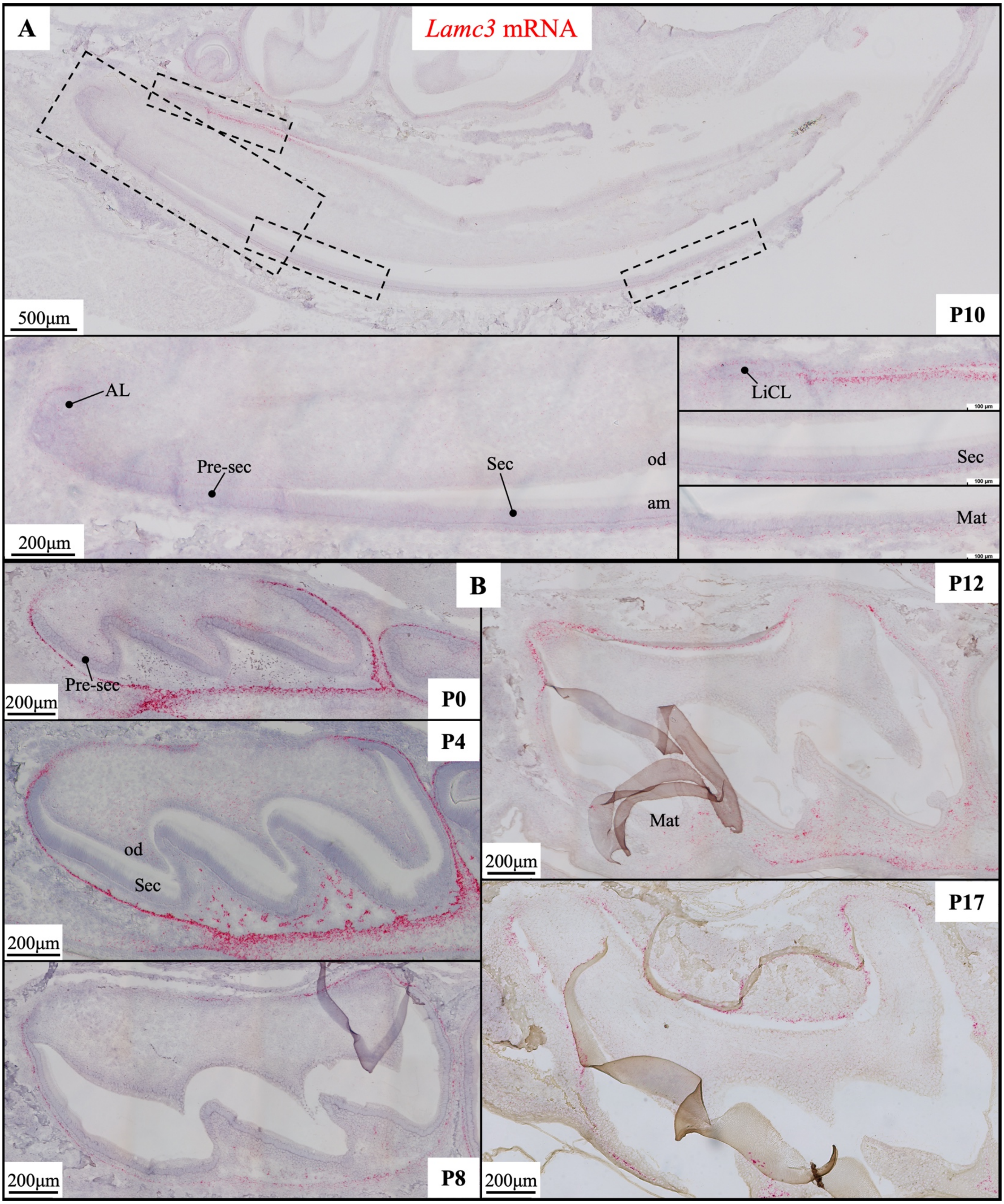

Fig S13. RNAscope in situ hybridization of *Lamc3* in mouse developing teeth. A. 10-day-old (P10) mandibular incisors that contain ameloblast developmental stages, including the dental epithelial stem cells in the apical loop (AL) that differentiate into pre-secretory (Pre-Sec), secretory (Sec), and maturation (Mat) stage ameloblasts (am). The lingual cervical loop (LiCL) is also shown. **B**. Newborn (P0), 4-day-old (P4), 8-day-old (P8), 12-day-old (P12), and 17-day-old (P17) maxillary 1st molars. Key: od, odontoblasts.

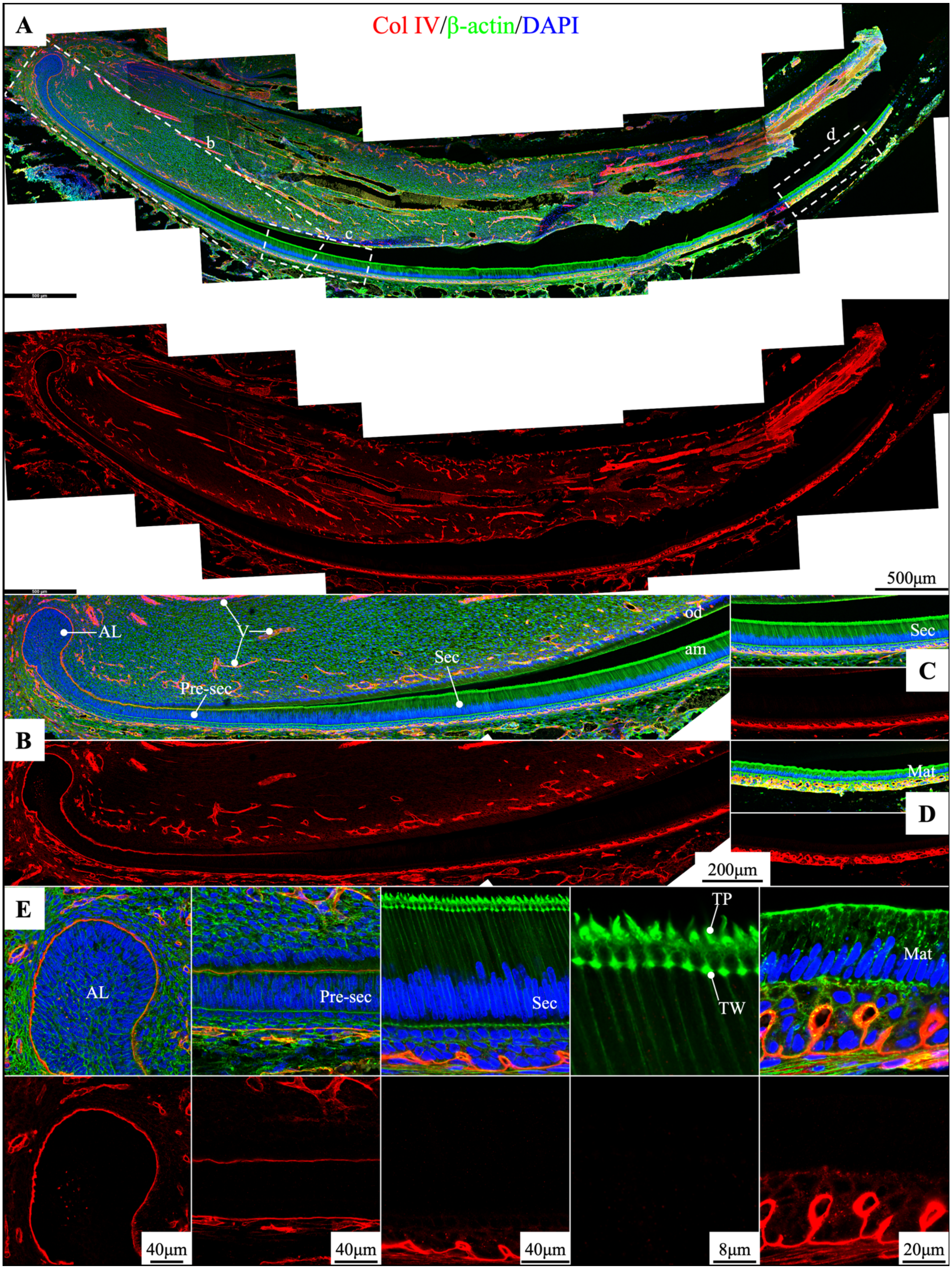

Fig S14. Immunohistochemistry of type IV collagen in 10-day-old mouse mandibular incisors. Signal for type IV collagen (COL IV) is red. β-actin signal for cytoskeleton is green. DAPI for nuclei is blue. Mouse continuously growing incisors contain all developmental stages of ameloblasts (am) (**A**), from the dental epithelial stem cells in the apical loop (AL), to pre-secretory (Pre-Sec), secretory (Sec) stage ameloblasts (**B&C**), and further into maturation (Mat) stage ameloblasts (**D**). High magnification images are shown in **E**. Key: od, odontoblasts; V, blood vessels; TP, Tomes' process; TW, (distal) terminal web.

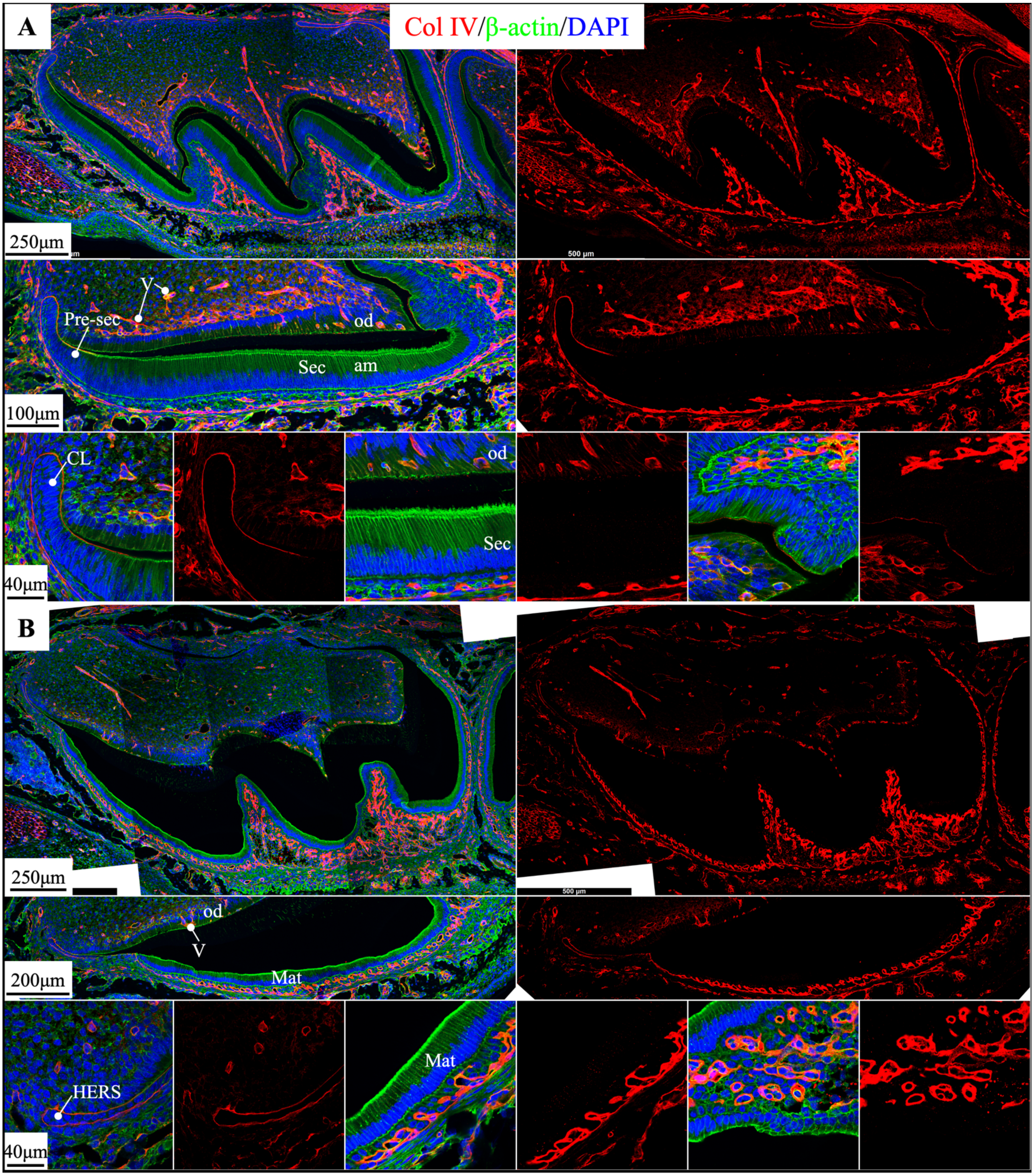

Fig S15. Immunohistochemistry of type IV collagen in 4- (A) and 10-day-old (B) mouse maxillary 1st molars. Signal for type IV collagen (COL IV) is red. β-actin signal for cytoskeleton is green. DAPI for nuclei is blue. **A**. The majority of the 4-day-old enamel organ epithelium is in the secretory (Sec) stage, except for the cervical loop (CL) and pre-secretory stage ameloblasts (Pre-Sec) near the cervical loop. **B**. The majority of the 10-day-old enamel organ epithelium is in the maturation (Mat) stage. Hertwig's epithelial root sheath (HERS) is observed at the root aspect of the developing teeth. Key: am, ameloblasts; od, odontoblasts; V, blood vessels.

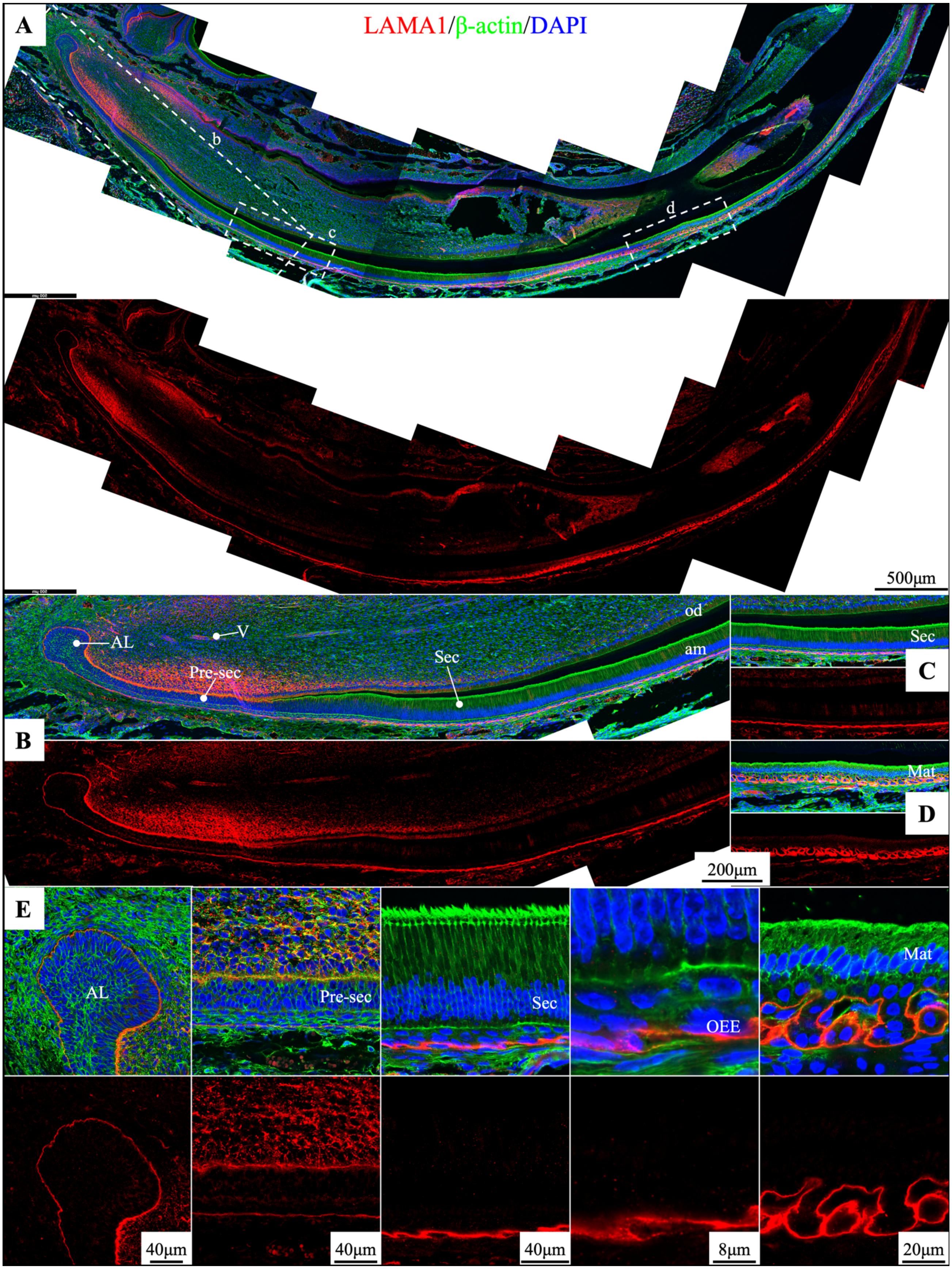

Fig S16. Immunohistochemistry of LAMA1 in 10-day-old mouse mandibular incisors. Signal for LAMA1 is red. β-actin signal for cytoskeleton is green. DAPI for nuclei is blue. Mouse continuously growing incisors contain all developmental stages of ameloblasts (am) (**A**), from the dental epithelial stem cells in the apical loop (AL) to pre-secretory (Pre-Sec), secretory (Sec) stages ameloblasts (**B&C**), and further into maturation (Mat) stage ameloblasts (**D**). High magnification images are shown in **E**. Key: od, odontoblasts; V, blood vessels; OEE, outer enamel epithelium.

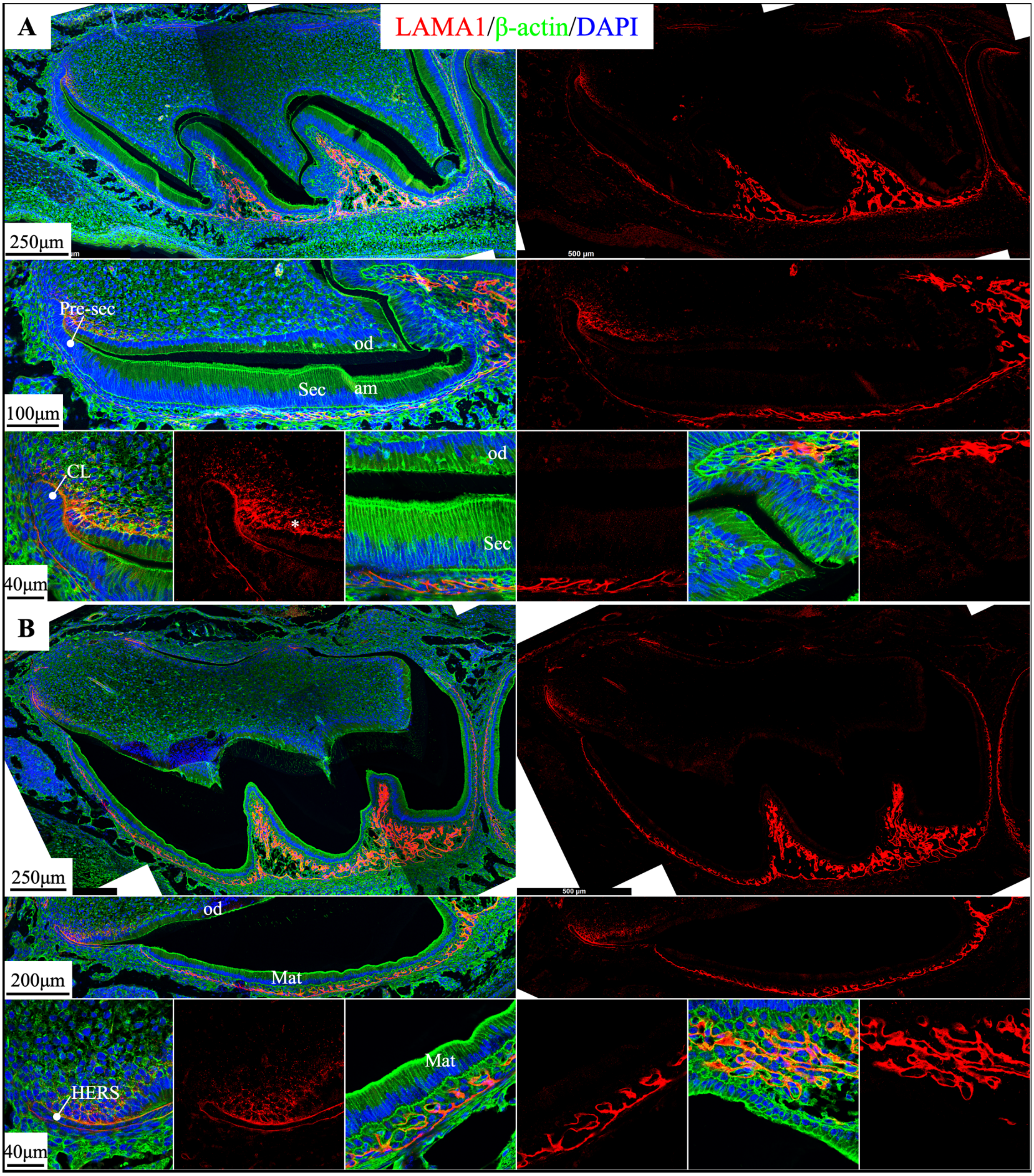

Fig S17. Immunohistochemistry of LAMA1 in 4- (A) and 10-day-old (B) mouse maxillary 1st molars. Signal for LAMA1 is red. β-actin signal for cytoskeleton is green. DAPI for nuclei is blue. **A**. The majority of the 4-day-old enamel organ epithelium is in the secretory (Sec) stage, except for the cervical loop (CL) and pre-secretory stage ameloblasts (Pre-Sec) near the cervical loop. The extracellular matrix of apical dental papilla cells is marked by asterisk (*). **B**. The majority of the 10-day-old enamel organ epithelium is in the maturation (Mat) stage. Hertwig's epithelial root sheath (HERS) is observed at the root aspect of the developing teeth. Key: am, ameloblasts; od, odontoblasts; V, blood vessels.

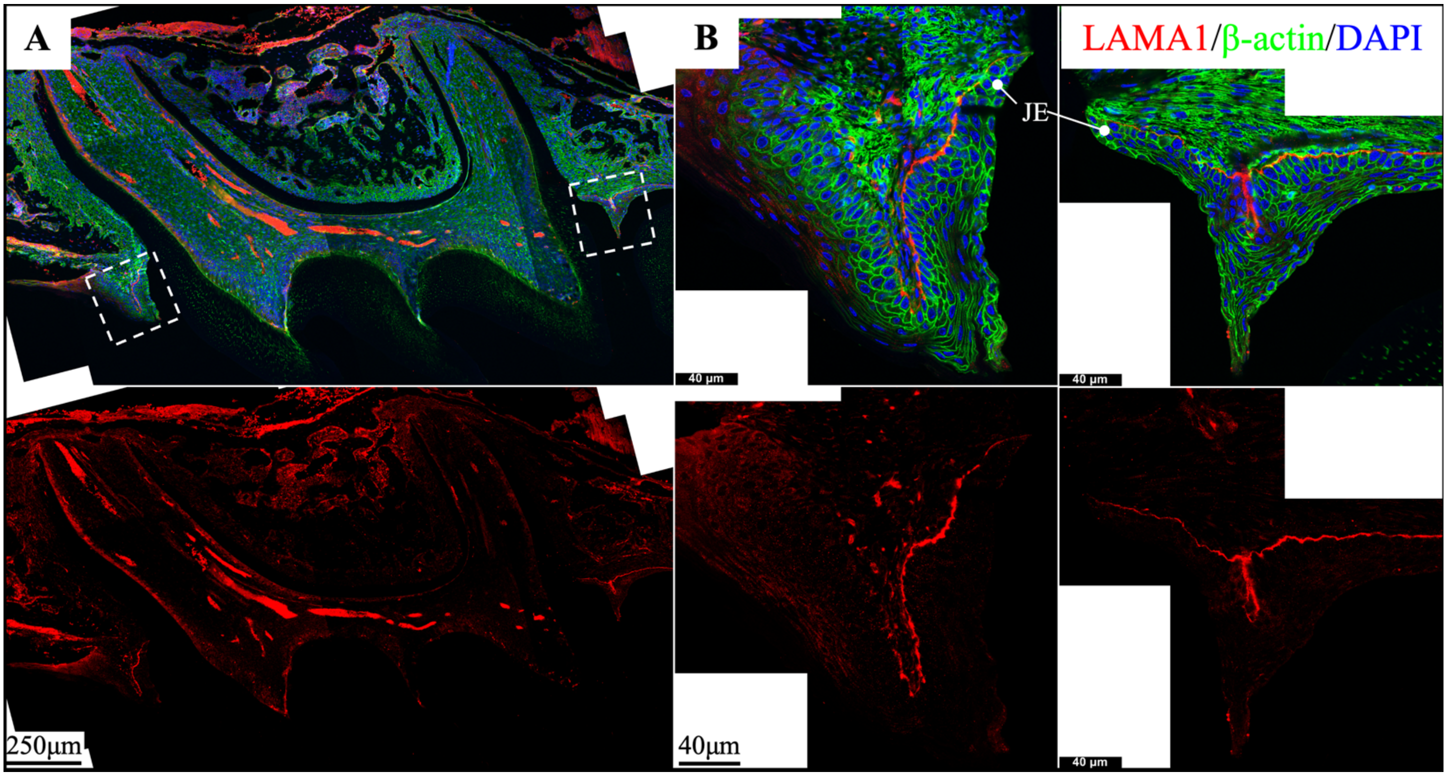

Fig S18. Immunohistochemistry of LAMA1 in 21-day-old mouse maxillary 1st molars. Signal for LAMA1 is red. β-actin signal for cytoskeleton is green. DAPI for nuclei is blue. **A**. The molar has erupted, leaving two sites of junctional epithelium (JE), mesial and distal to the tooth on this section. **B**. High magnification images of junctional epithelium.

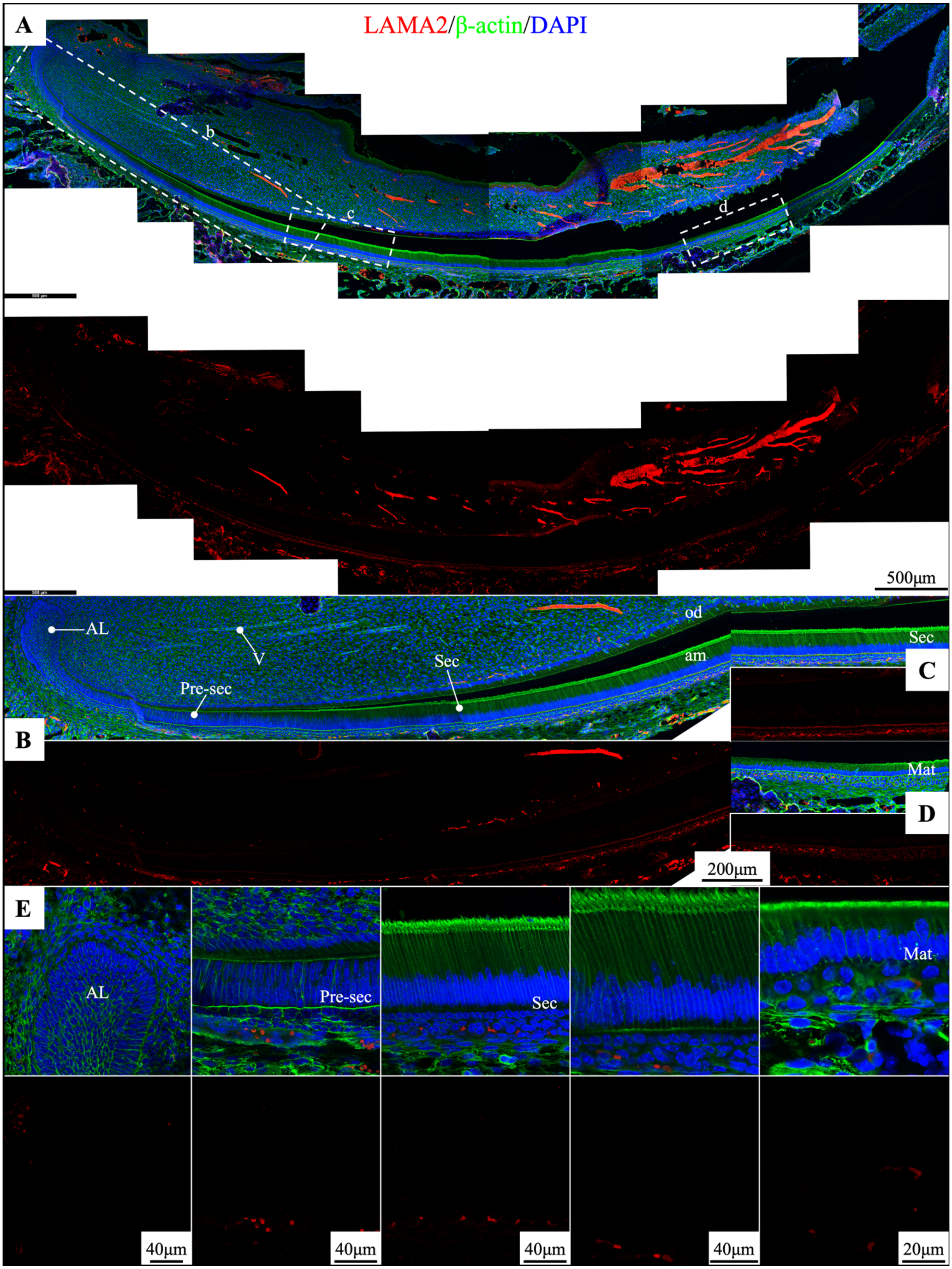

Fig S19. Immunohistochemistry of LAMA2 in 10-day-old mouse mandibular incisors. Signal for LAMA2 is red. β-actin signal for cytoskeleton is green. DAPI for nuclei is blue. Mouse continuously growing incisors contain all developmental stages of ameloblasts (am) (**A**), from the dental epithelial stem cells in the apical loop (AL) to pre-secretory (Pre-Sec), secretory (Sec) (**B&C**), and further into maturation (Mat) stage ameloblasts (**D**). High magnification images are shown in **E**. Key: od, odontoblasts.

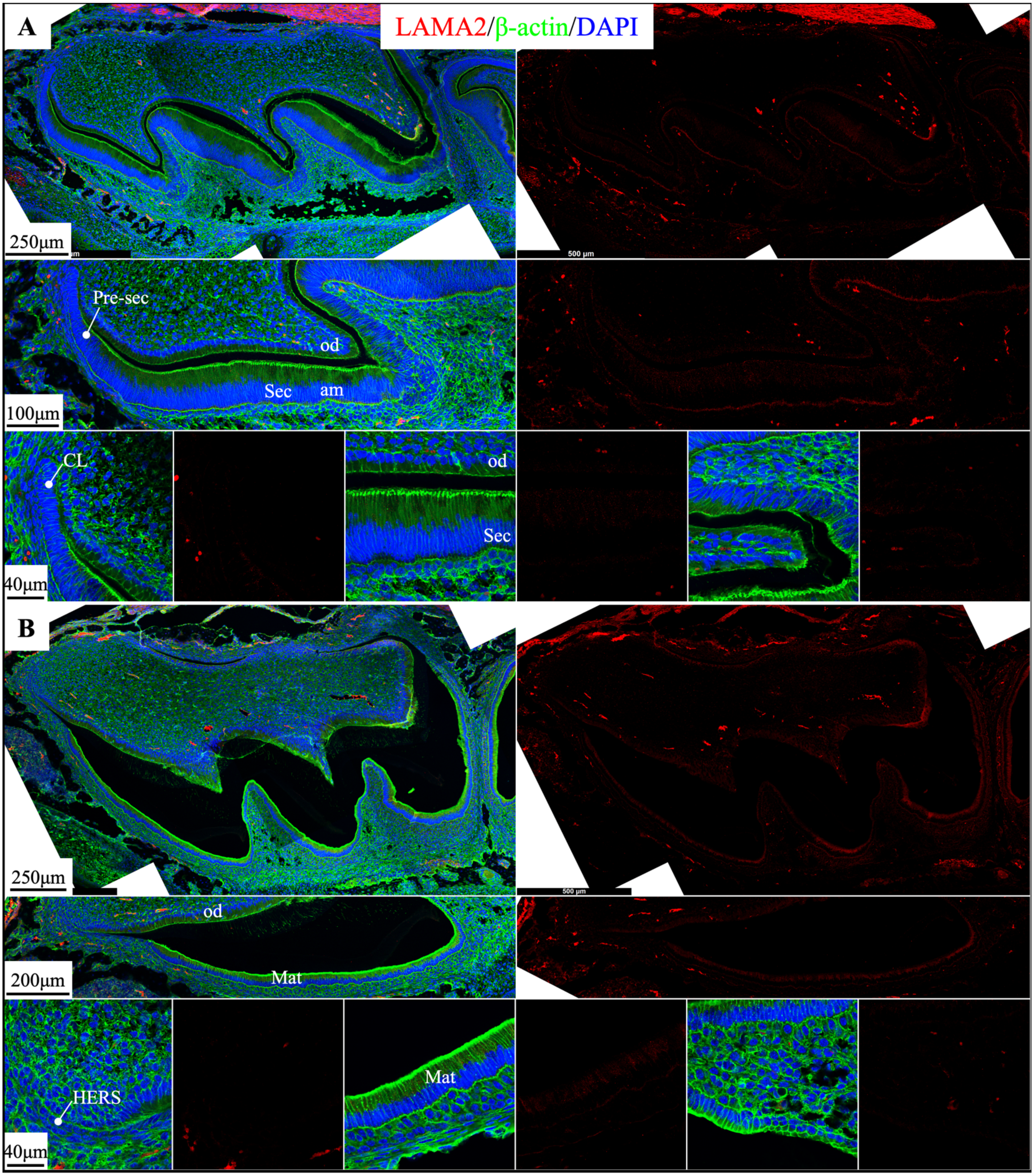

Fig S20. Immunohistochemistry of LAMA2 in 4- (A) and 10-day-old (B) mouse maxillary 1st molars. Signal for LAMA2 is red. β-actin signal for cytoskeleton is green. DAPI for nuclei is blue. **A**. The majority of the 4-day-old enamel organ epithelium is in the secretory (Sec) stage, except for the cervical loop (CL) and pre-secretory ameloblasts (Pre-Sec) near the cervical loop. **B**. The majority of the 10-day-old enamel organ epithelium is in the maturation (Mat) stage. Hertwig's epithelial root sheath (HERS) is observed at the root aspect of the developing teeth. Key: am, ameloblasts; od, odontoblasts.

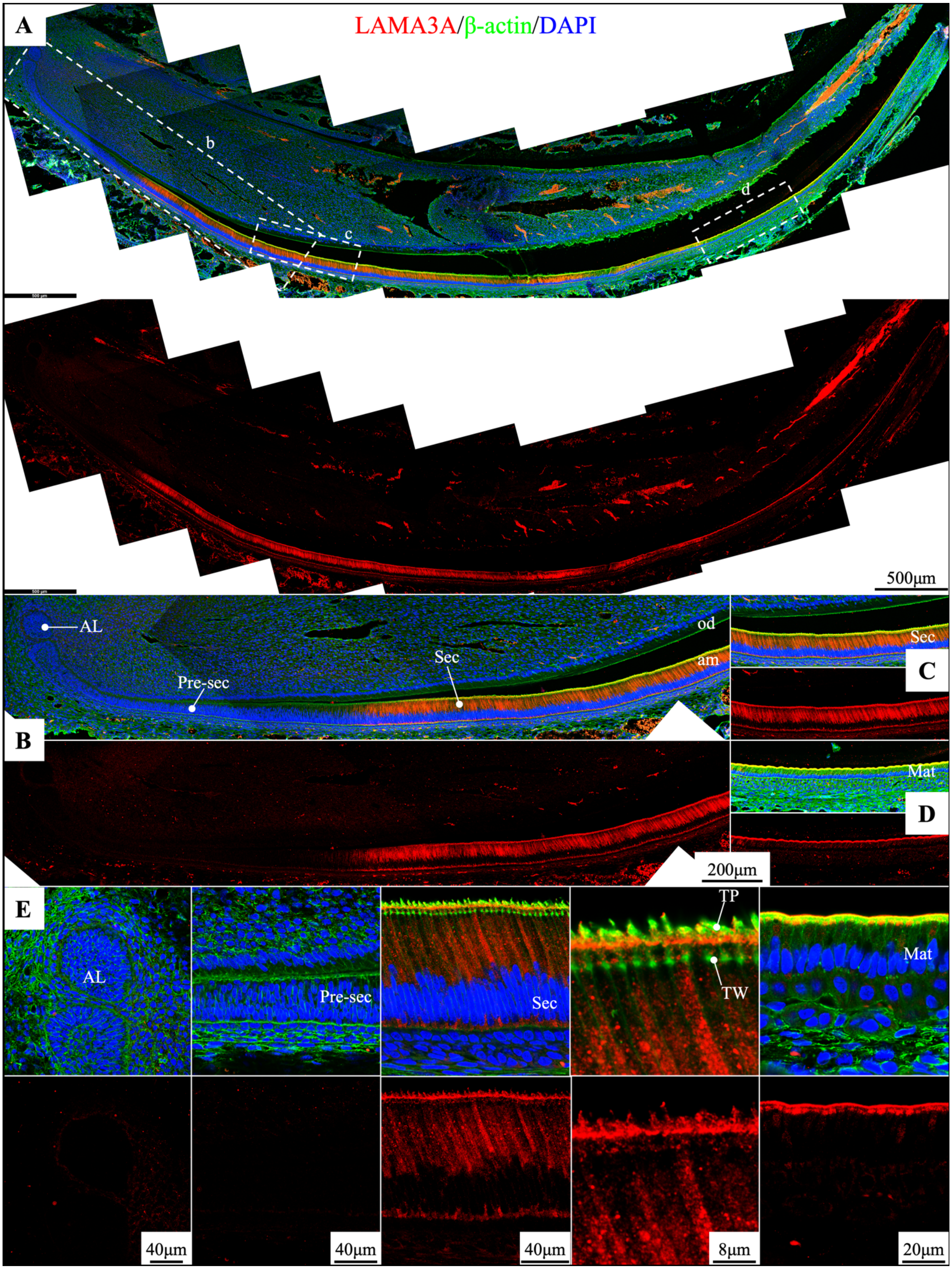

Fig S21. Immunohistochemistry of LAMA3A in 10-day-old mouse mandibular incisors. Signal for LAMA3A is red. β-actin signal for cytoskeleton is green. DAPI for nuclei is blue. Mouse continuously growing incisors contain all developmental stages of ameloblasts (am) (**A**), from the dental epithelial stem cells in the apical loop (AL) to pre-secretory (Pre-Sec), secretory (Sec) (**B&C**), and further into maturation (Mat) stage ameloblasts (**D**). High magnification images are shown in **E**. Key: od, odontoblasts; TP, Tomes' process; TW, (distal) terminal web.

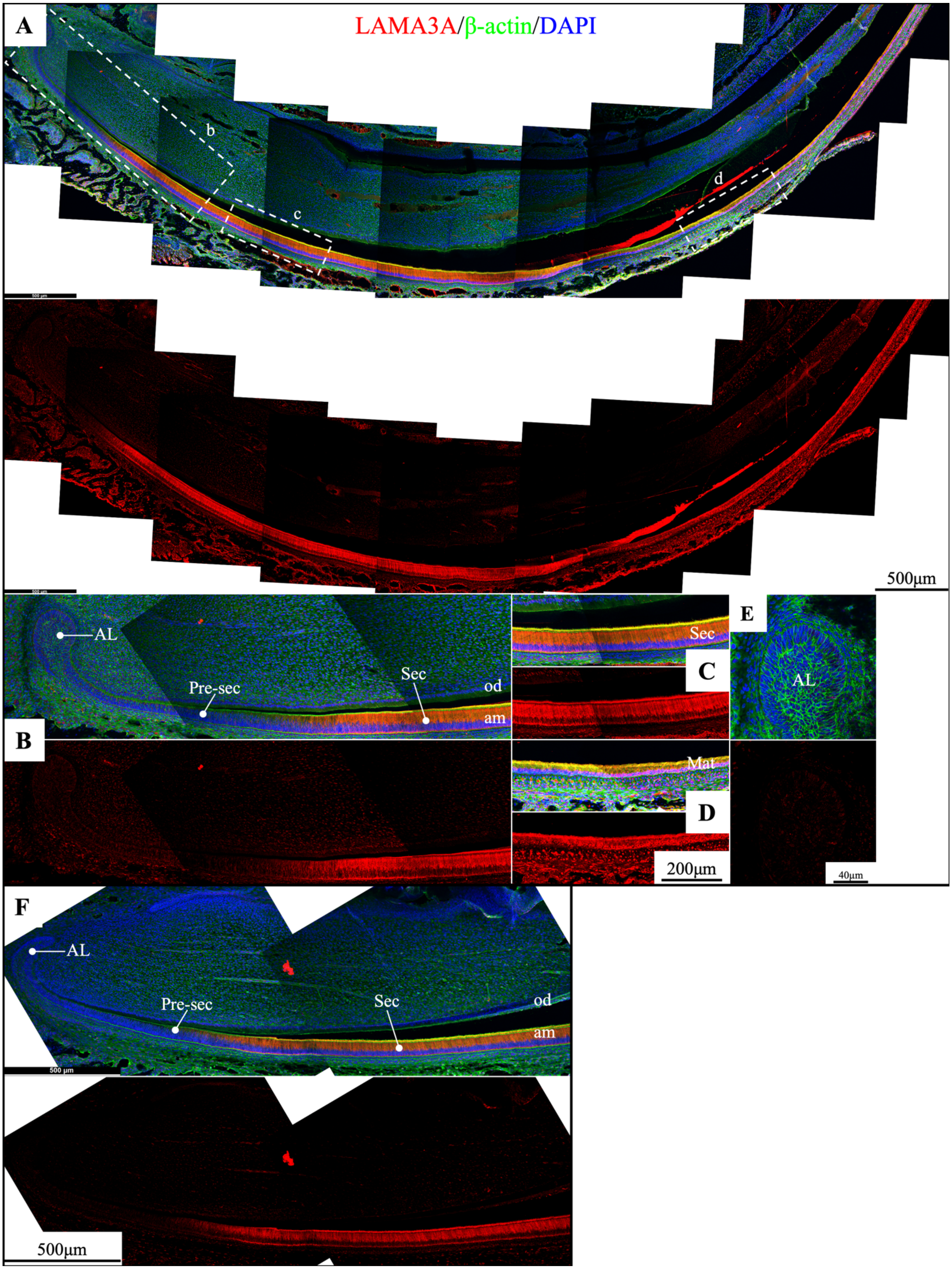

Fig S22. Immunohistochemistry of LAMA3A in 10-day-old mouse mandibular incisors. Signal for LAMA3A is red. β-actin signal for cytoskeleton is green. DAPI for nuclei is blue. Mouse continuously growing incisors contain all developmental stages of ameloblasts (am) (**A**), from the dental epithelial stem cells in the apical loop (AL), to pre-secretory (Pre-Sec), secretory (Sec) (**B&C**), and further into maturation (Mat) stage ameloblasts (**D**). High magnification image of cervical loop is shown in **E**. **F**. The apical region of another 10-day-old mouse mandibular incisor. Key: od, odontoblasts.

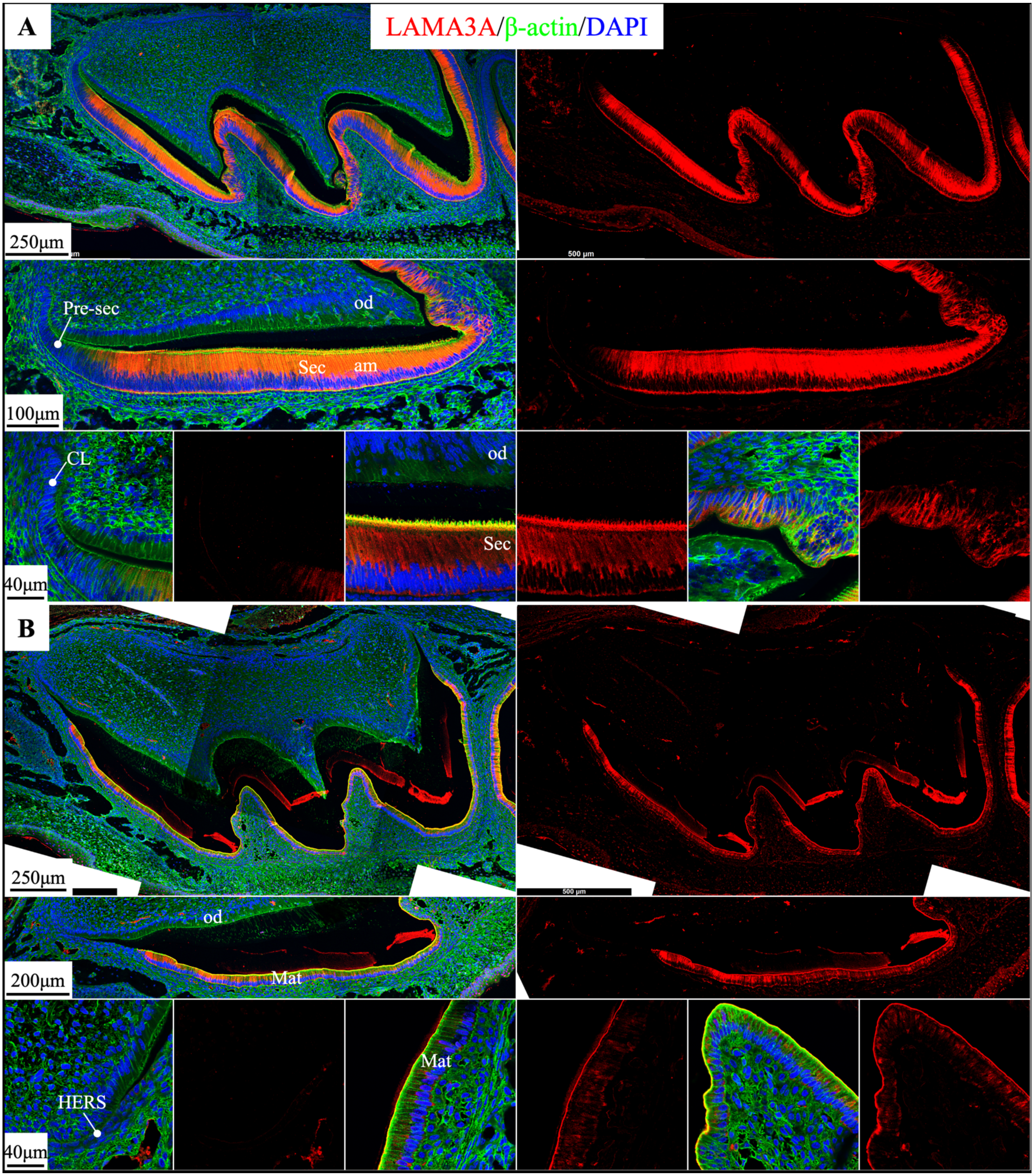

Fig S23. Immunohistochemistry of LAMA3A in 4- (A) and 10-day-old (B) mouse maxillary 1st molars. Signal for LAMA3A is in red. β-actin signal for cytoskeleton is green. DAPI for nuclei is blue. **A**. The majority of the 4-day-old enamel organ epithelium is in the secretory (Sec) stage, except for the cervical loop (CL) and the pre-secretory stage ameloblasts (Pre-Sec) near the cervical loop. **B**. The majority of the 10-day-old enamel organ epithelium is in the maturation (Mat) stage. Hertwig's epithelial root sheath (HERS) is observed at the root aspect of the developing teeth. Key: am, ameloblasts; od, odontoblasts.

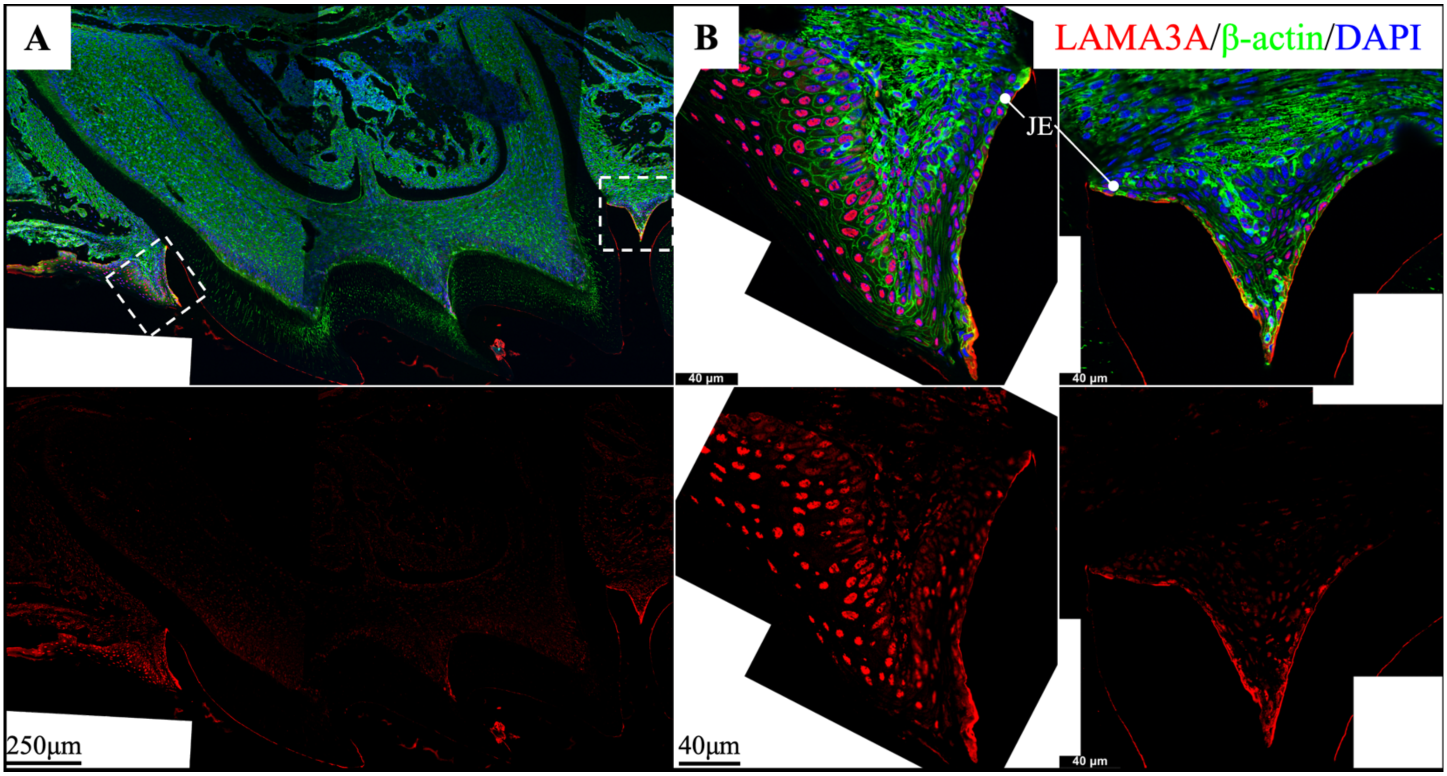

Fig S24. Immunohistochemistry of LAMA3A in 21-day-old mouse maxillary 1st molars. Signal for LAMA3A is red. β-actin signal for cytoskeleton is green. DAPI for nuclei is blue. **A**. The molar has erupted, leaving two sites of junctional epithelium (JE) mesial and distal to the tooth on this section (dashed boxes). **B**. High magnification images of junctional epithelium. Non-specific signal is observed in some nuclei.

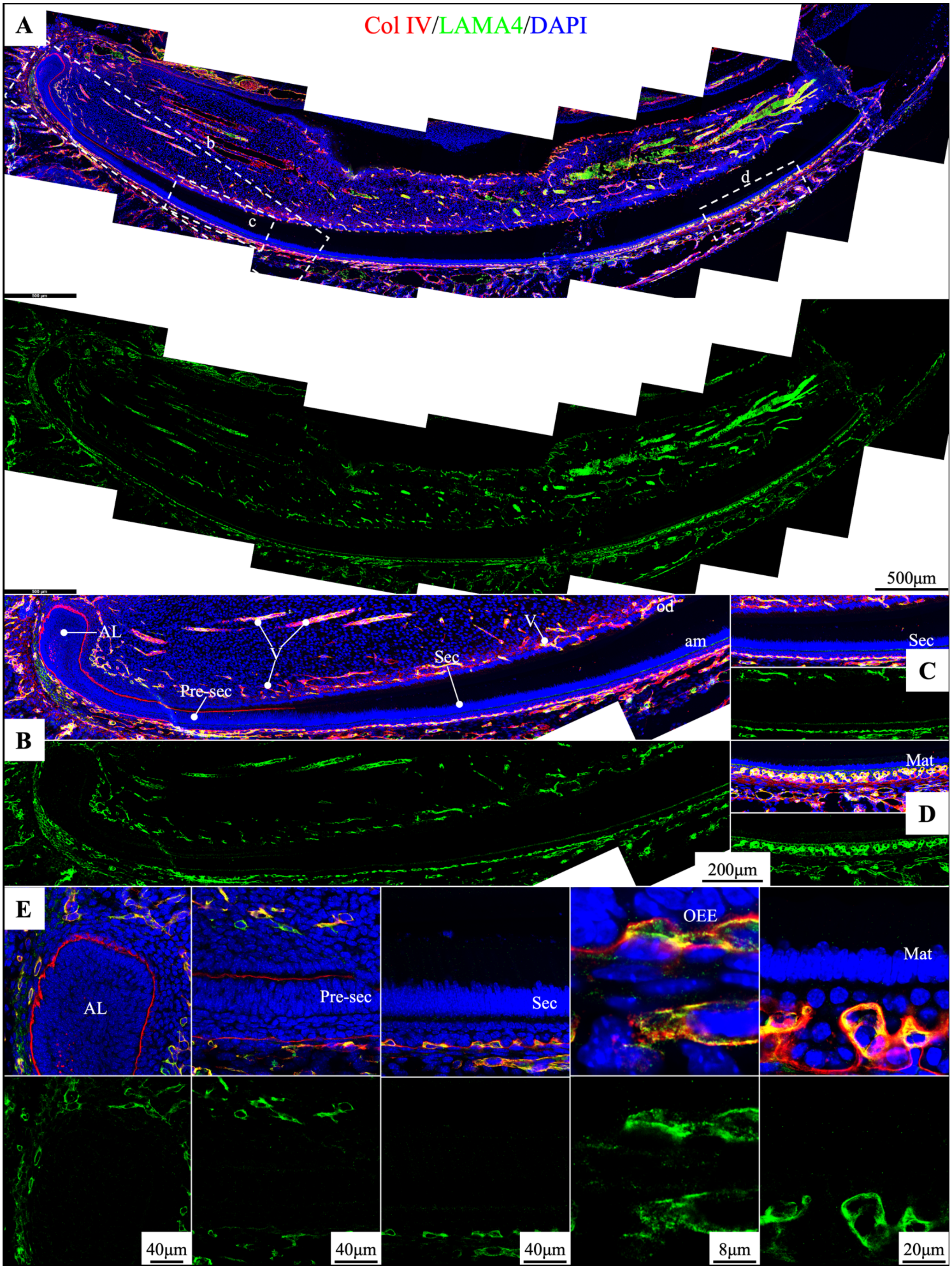

Fig S25. Immunohistochemistry of LAMA4 in 10-day-old mouse mandibular incisors. Signal for type IV collagen is red. LAMA4 signal is green. DAPI for nuclei is blue. Mouse continuously growing incisors contain all developmental stages of ameloblasts (am) (**A**), from the dental epithelial stem cells in the apical loop (AL) to pre-secretory (Pre-Sec), secretory (Sec) (**B&C**), and further into maturation (Mat) stage ameloblasts (**D**). High magnification images are shown in **E**. Key: od, odontoblasts; V, blood vessels; OEE, outer enamel epithelium.

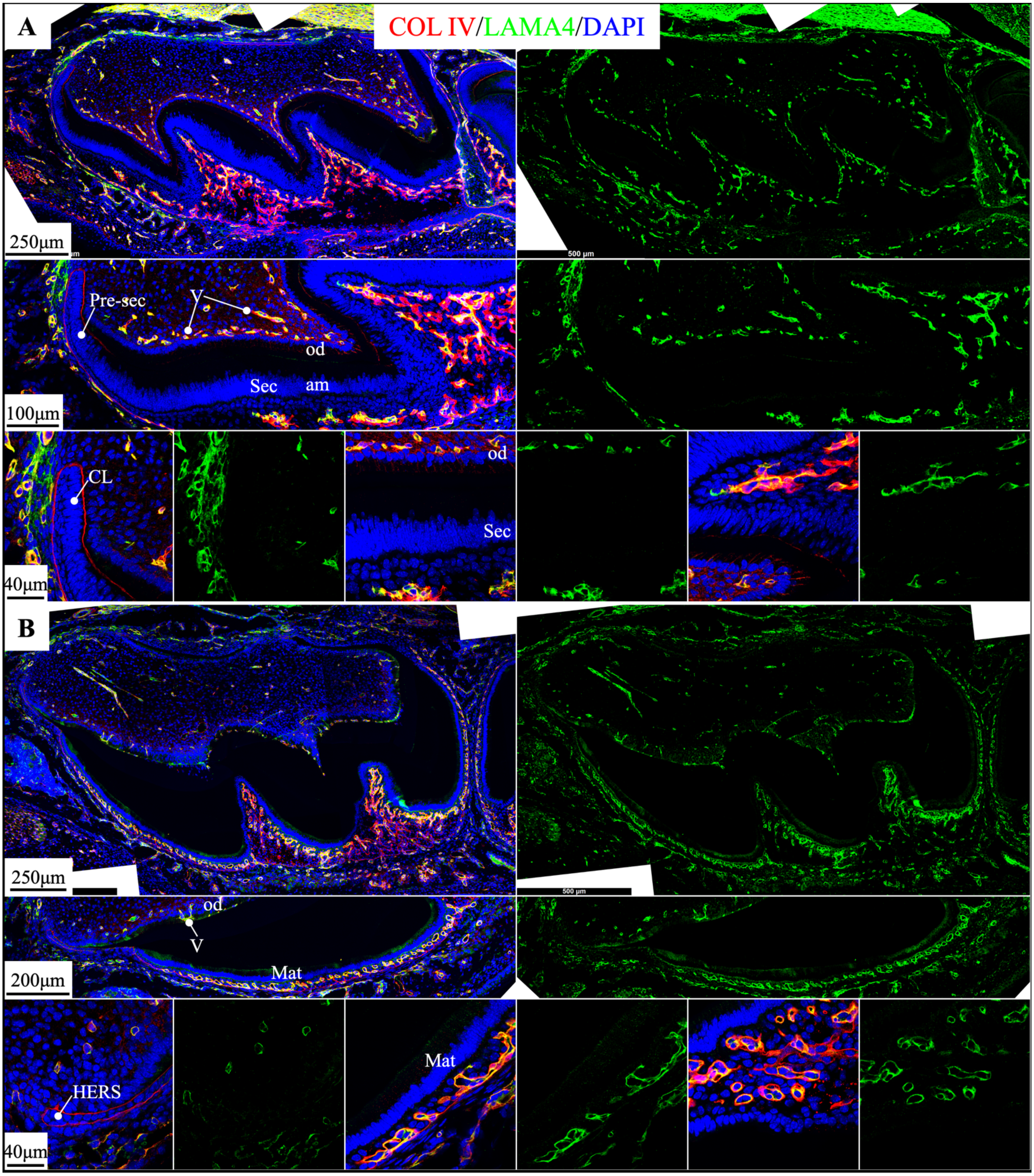

Fig S26. Immunohistochemistry of LAMA4 in 4- (A) and 10-day-old (B) mouse maxillary 1st molars. Signal for type IV collagen is red, LAMA4 signal is green. DAPI for nuclei is blue. **A**. The majority of the 4-day-old enamel organ epithelium is in the secretory (Sec) stage, except for the cervical loop (CL) and pre-secretory (Pre-Sec) stage ameloblasts near the cervical loop. **B**. The majority of the 10-day-old enamel organ epithelium is in the maturation (Mat) stage. Hertwig's epithelial root sheath (HERS) is observed at the root aspect of the developing teeth. Key: am, ameloblasts; od, odontoblasts; V, blood vessels.

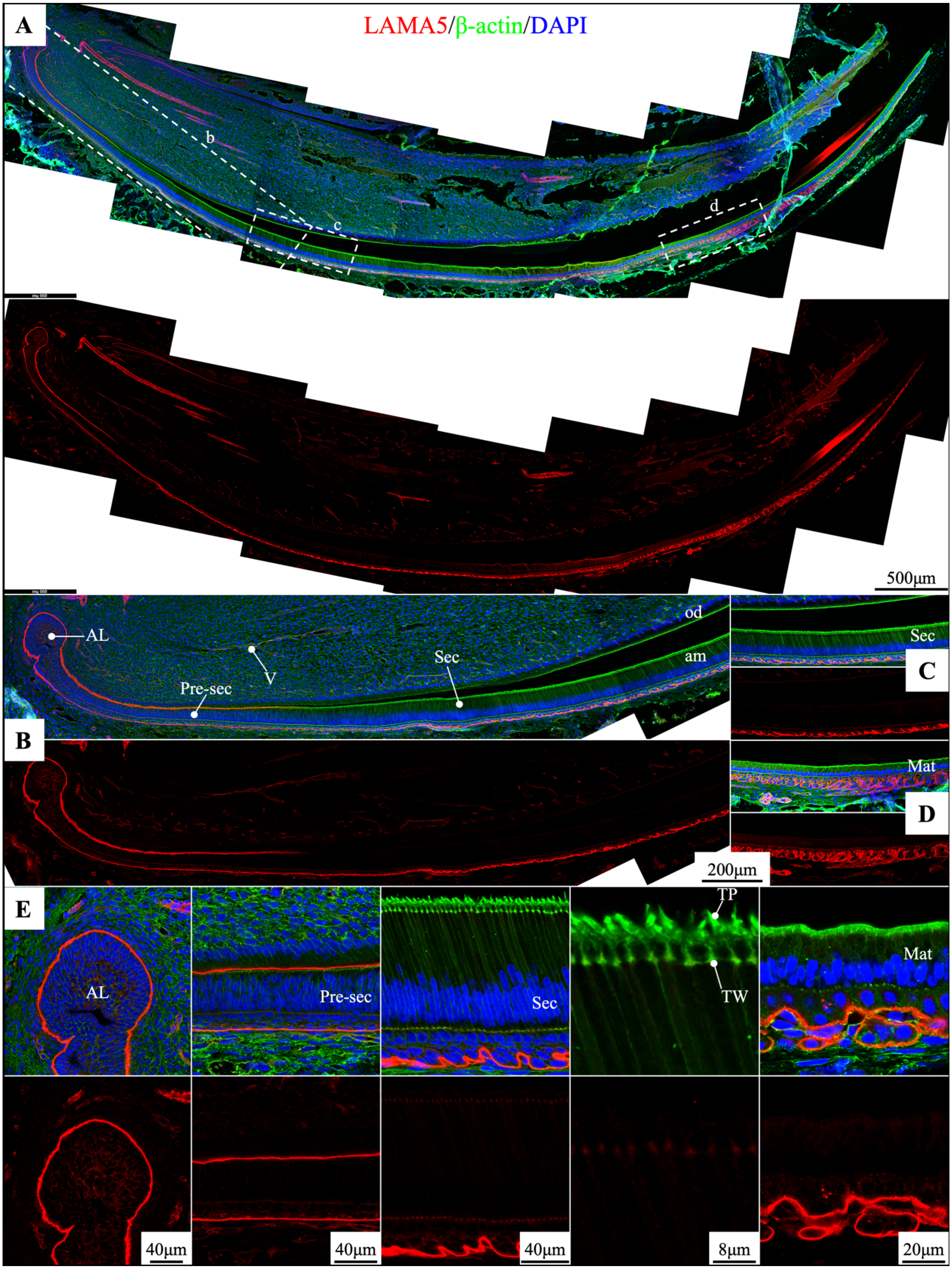

Fig S27. Immunohistochemistry of LAMA5 in 10-day-old mouse mandibular incisors. Signal for LAMA5 is red. β-actin signal for cytoskeleton is green. DAPI for nuclei is blue. Mouse continuously growing incisors contain all developmental stages of ameloblasts (am) (**A**), from the dental epithelial stem cells in the apical loop (AL), to pre-secretory (Pre-Sec), secretory (Sec) (**B&C**), and further into maturation (Mat) stages ameloblasts (**D**). High magnification images are shown in **E**. Key: od, odontoblasts; TP, Tomes' process; TW, (distal) terminal web; V, blood vessels.

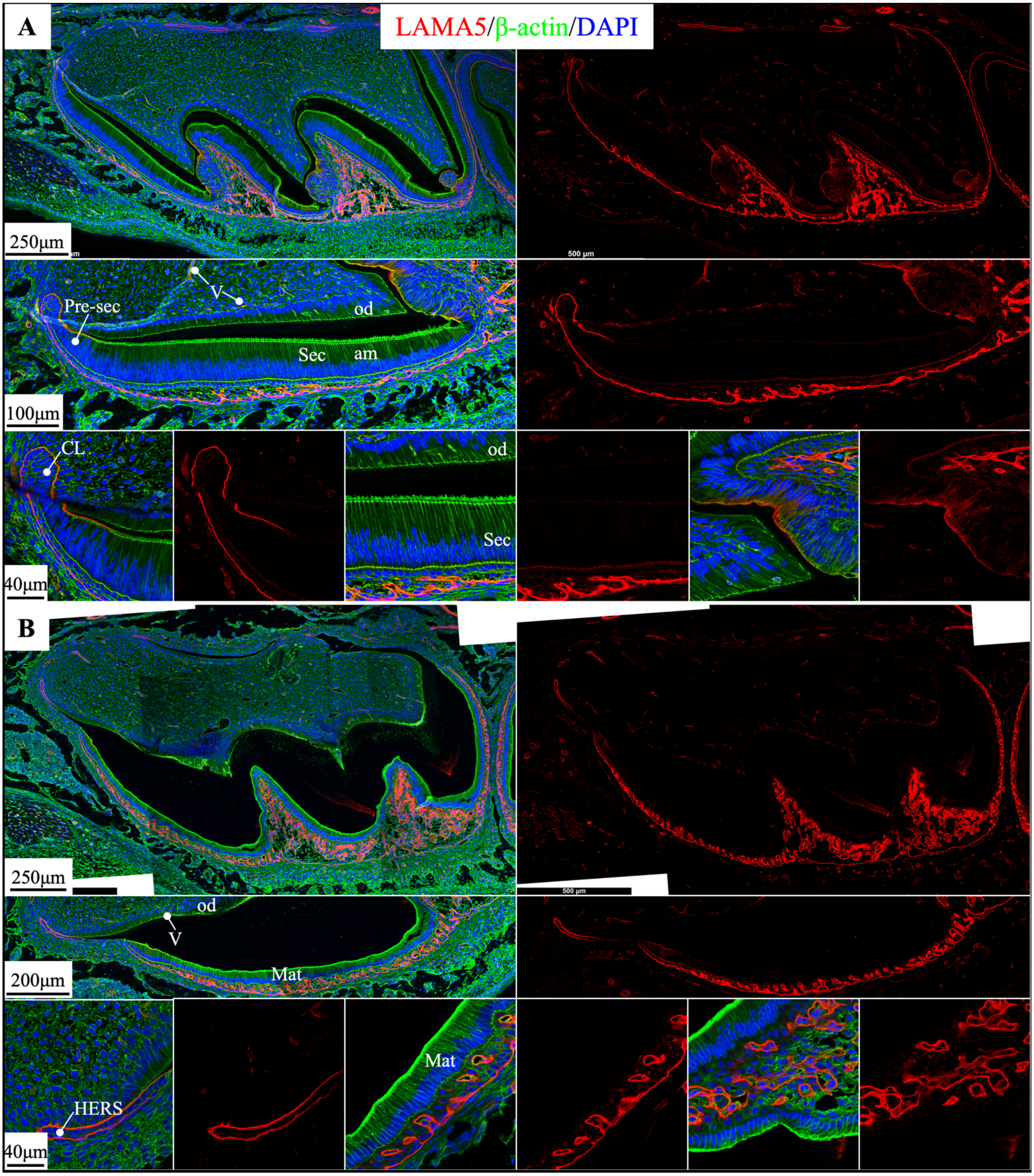

Fig S28. Immunohistochemistry of LAMA5 in 4- (A) and 10-day-old (B) mouse maxillary 1st molars. Signal for LAMA5 is red. β-actin signal for cytoskeleton is green. DAPI for nuclei is blue. **A**. The majority of the 4-day-old enamel organ epithelium is in the secretory (Sec) stage, except for the cervical loop (CL) and the pre-secretory (Pre-Sec) stage ameloblasts near the cervical loop. **B**. The majority of the 10-day-old enamel organ epithelium is in the maturation (Mat) stage. Hertwig's epithelial root sheath (HERS) is observed at the root aspect of the developing teeth. Key: am, ameloblasts; od, odontoblasts; V, blood vessels.

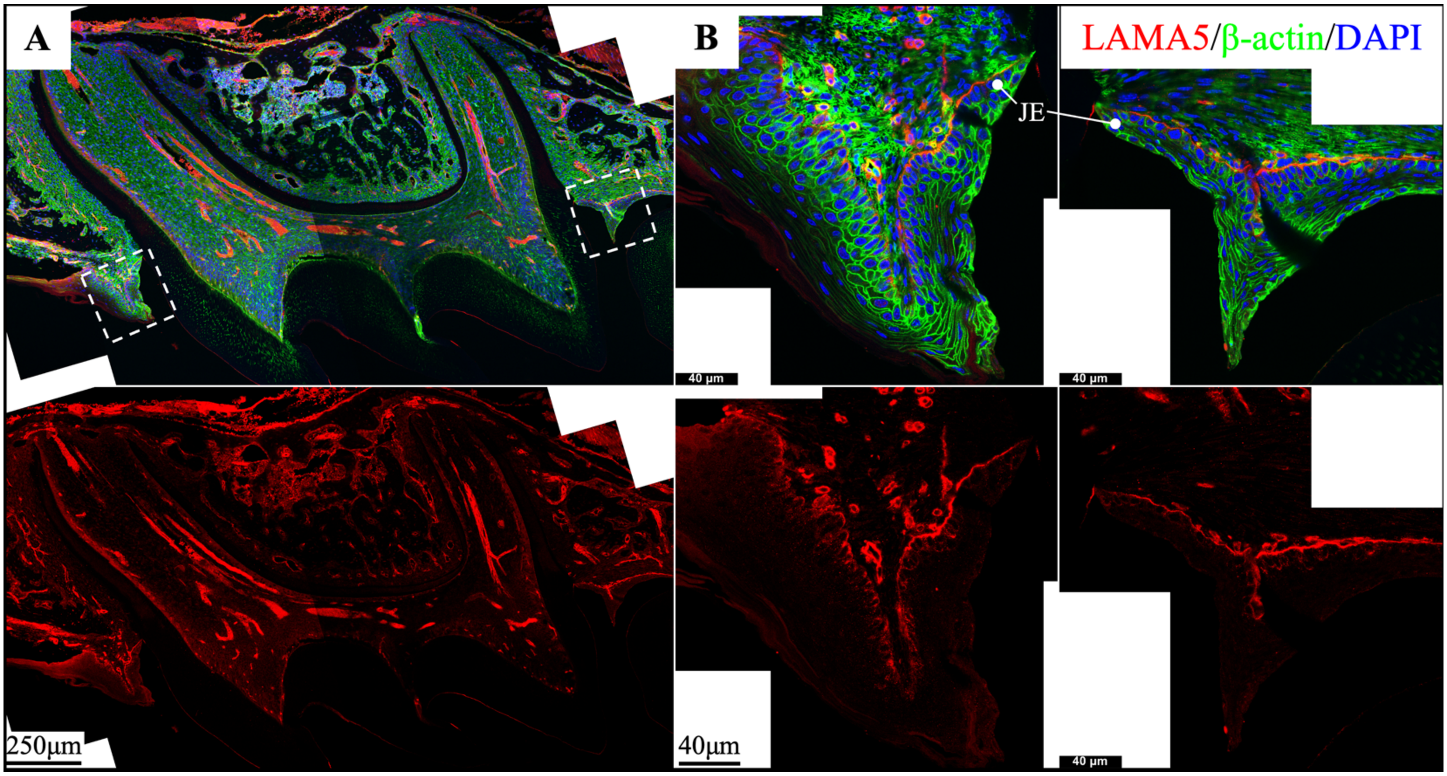

Fig S29. Immunohistochemistry of LAMA5 in 21-day-old mouse maxillary 1st molars. Signal for LAMA5 is red. β-actin signal for cytoskeleton are green. DAPI for nuclei is blue. **A**. The molar has erupted, leaving two sites of junctional epithelium (JE) mesial and distal to the tooth (dashed boxes) on this section. **B**. High magnification images of junctional epithelium.

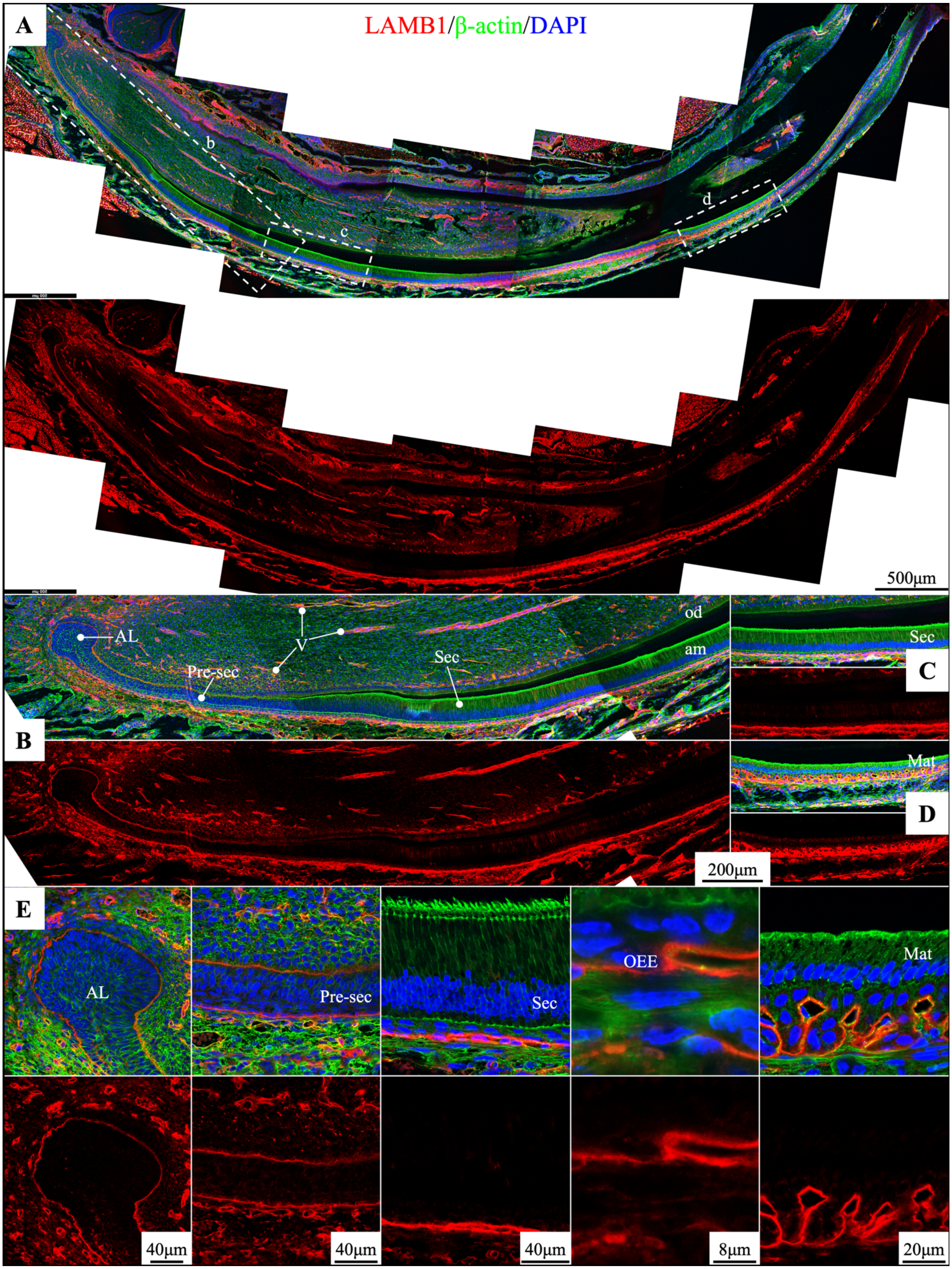

Fig S30. Immunohistochemistry of LAMB1 in 10-day-old mouse mandibular incisors. Signal for LAMB1 are in red. β-actin signal for cytoskeleton is green. DAPI for nuclei is blue. Mouse continuously growing incisors contain all developmental stages of ameloblasts (am) (**A**), from the dental epithelial stem cells in the apical loop (AL), to pre-secretory (Pre-Sec), secretory (Sec) (**B&C**), and further into maturation (Mat) stage ameloblasts (**D**). High magnification images are shown in **E**. Key: od, odontoblasts; OEE, outer enamel epithelium; V, blood vessels.

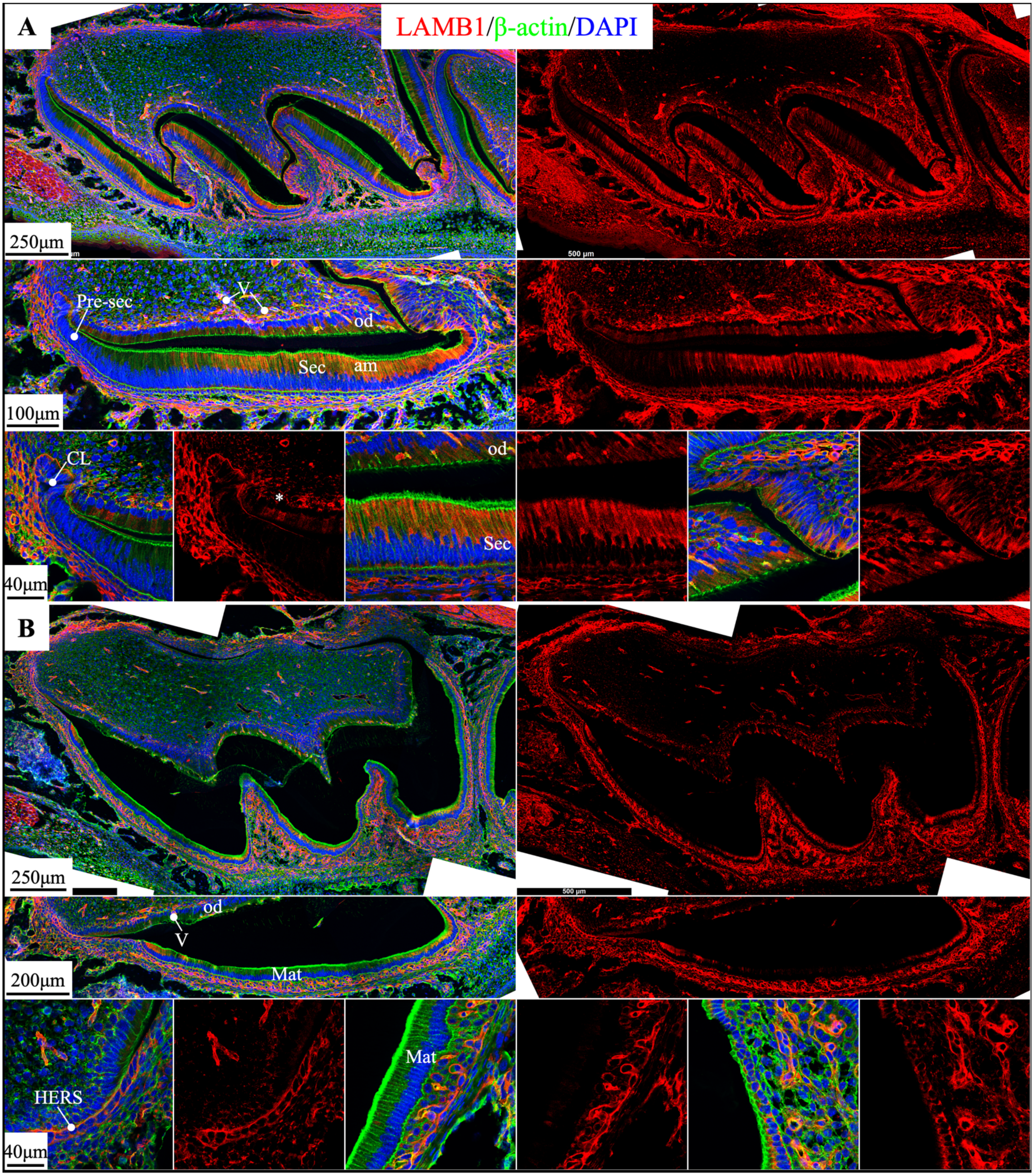

Fig S31. Immunohistochemistry of LAMB1 in 4- (A) and 10-day-old (B) mouse maxillary 1st molars. Signal for LAMB1 is red. β-actin signal for cytoskeleton is green. DAPI for nuclei is blue. **A**. The majority of the 4-day-old enamel organ epithelium is in the secretory (Sec) stage, except for the cervical loop (CL) and pre-secretory stage ameloblasts (Pre-Sec) near the cervical loop. The extracellular matrix of apical dental papilla cells is marked by an asterisk (*). **B**. The majority of the 10-day-old enamel organ epithelium is in the maturation (Mat) stage. The Hertwig's epithelial root sheath (HERS) is observed at the root aspect of the developing teeth. Key: am, ameloblasts; od, odontoblasts; V, blood vessels.

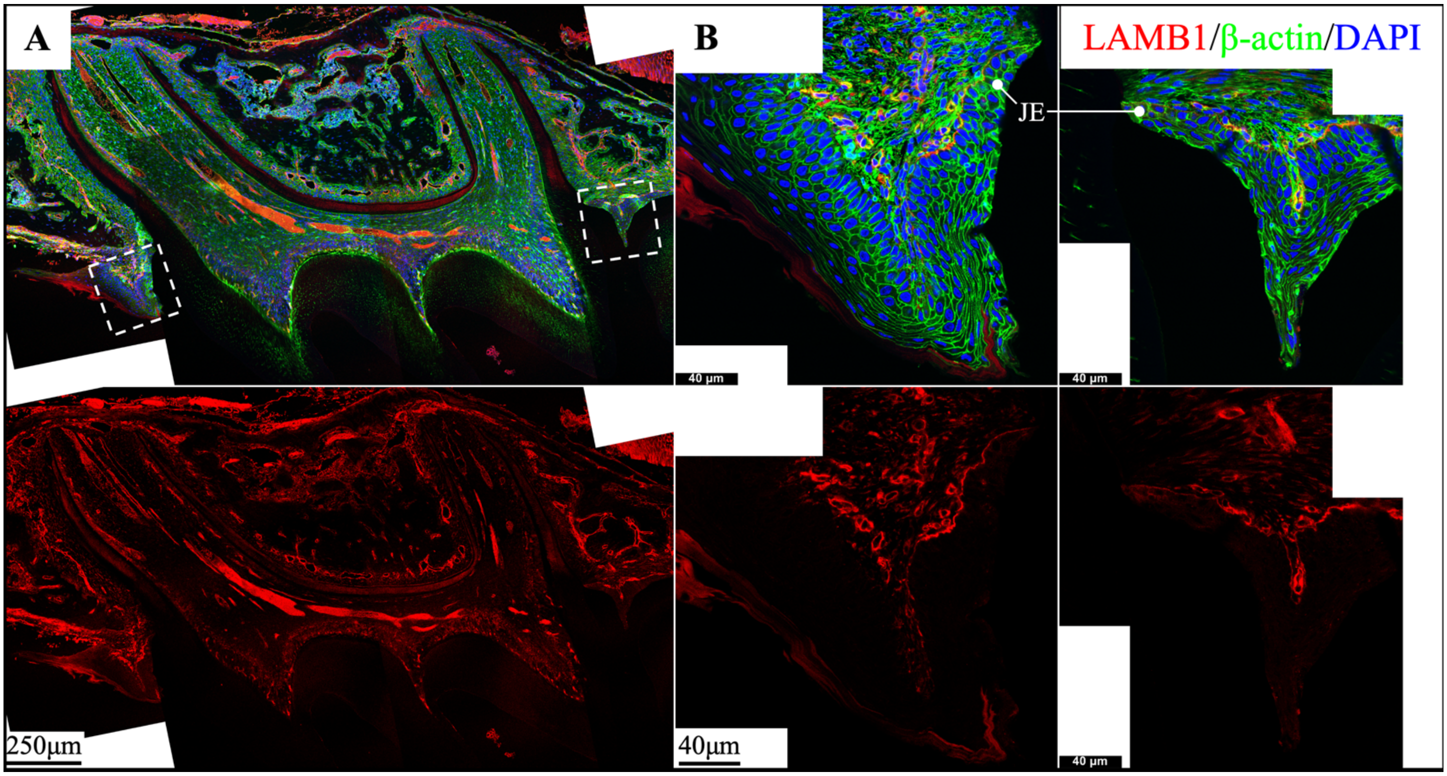

Fig S32. Immunohistochemistry of LAMB1 in 21-day-old mouse maxillary 1st molars. Signal for LAMB1 is red. β-actin signal for cytoskeleton is green. DAPI for nuclei is blue. **A**. The molar has erupted, leaving two sites of junctional epithelium (JE) mesial and distal to the tooth (dashed boxes) on this section. **B**. High magnification images of the junctional epithelium.

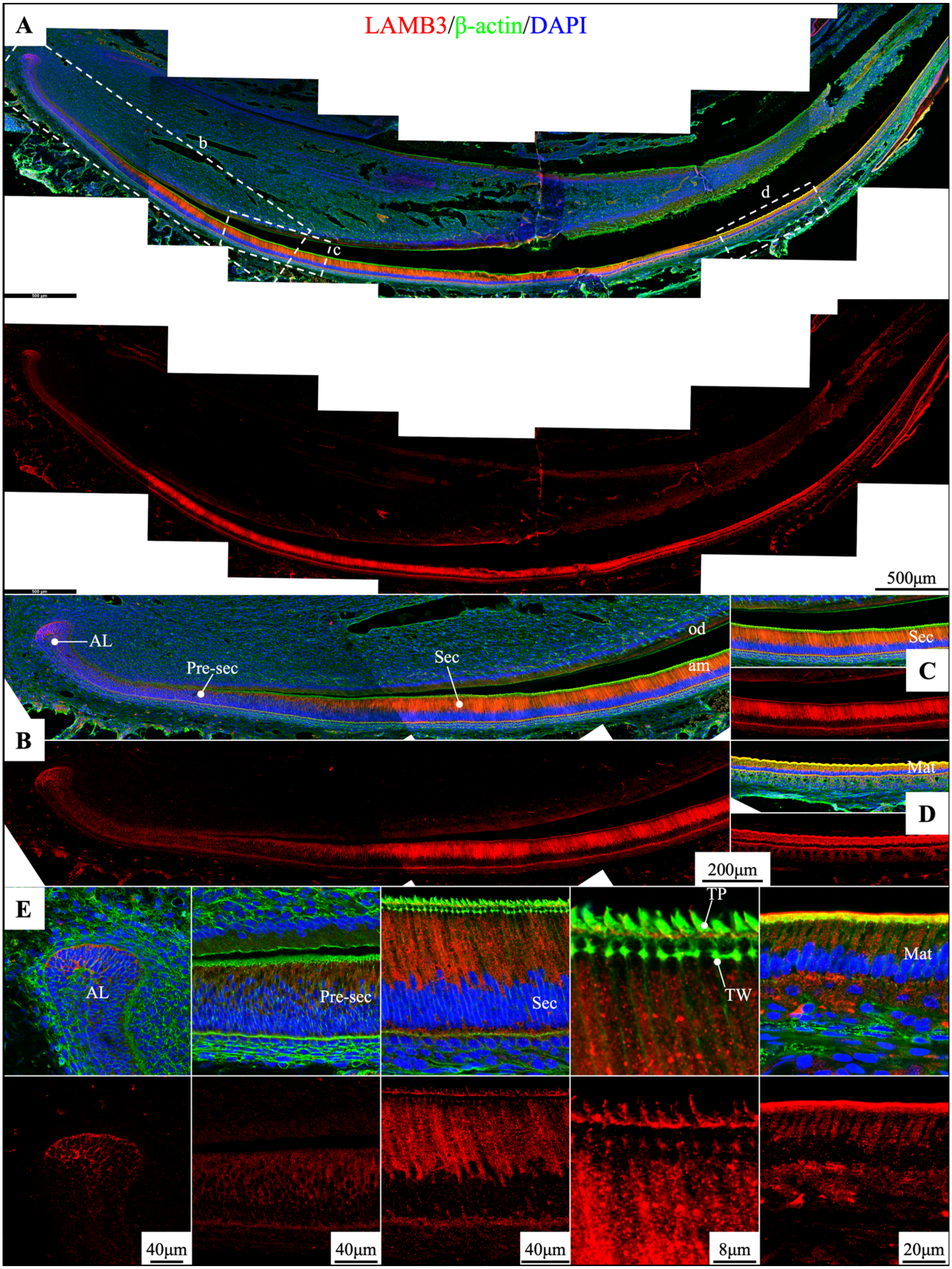

Fig S33. Immunohistochemistry of LAMB3 in 10-day-old mouse mandibular incisors. Signal for LAMB3 is red. β-actin signal for cytoskeleton is green. DAPI for nuclei is blue. Mouse continuously growing incisors contain all developmental stages of ameloblasts (am) (**A**), from the dental epithelial stem cells in the apical loop (AL), to pre-secretory (Pre-Sec), secretory (Sec) (**B&C**), and further into maturation (Mat) stage ameloblasts (**D**). High magnification images are shown in **E**. Key: od, odontoblasts; TP, Tomes' process; TW, (distal) terminal web.

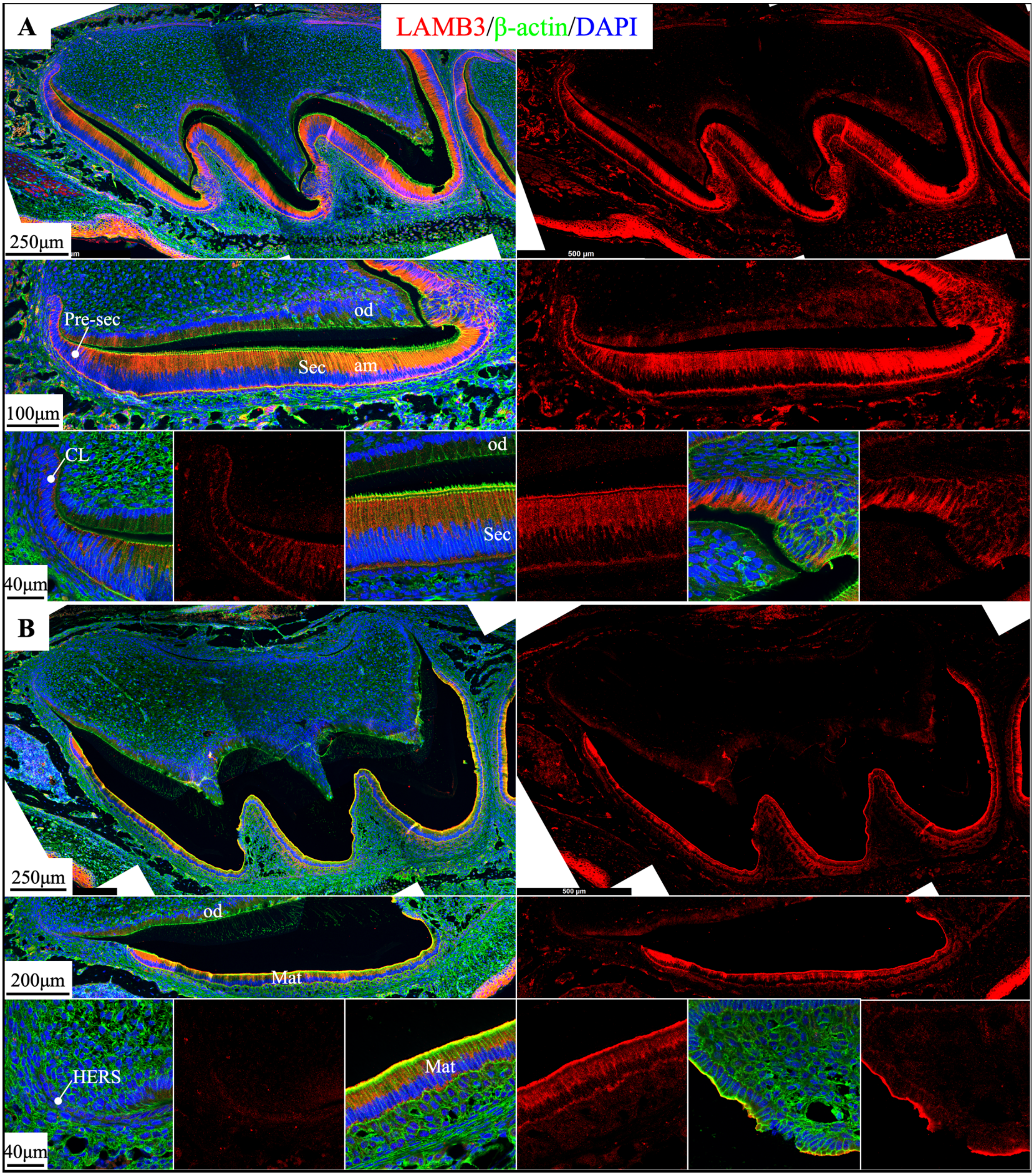

Fig S34. Immunohistochemistry of LAMB3 in 4- (A) and 10-day-old (B) mouse maxillary 1st molars. Signal for LAMB3 is red. β-actin signal for cytoskeleton is green. DAPI for nuclei is blue. **A**. The majority of the 4-day-old enamel organ epithelium is in the secretory (Sec) stage, except for the cervical loop (CL) and pre-secretory stage ameloblasts (Pre-Sec) near the cervical loop. **B**. The majority of the 10-day-old enamel organ epithelium is in the maturation (Mat) stage. Hertwig's epithelial root sheath (HERS) is observed at the root aspect of the developing teeth. Key: am, ameloblasts; od, odontoblasts.

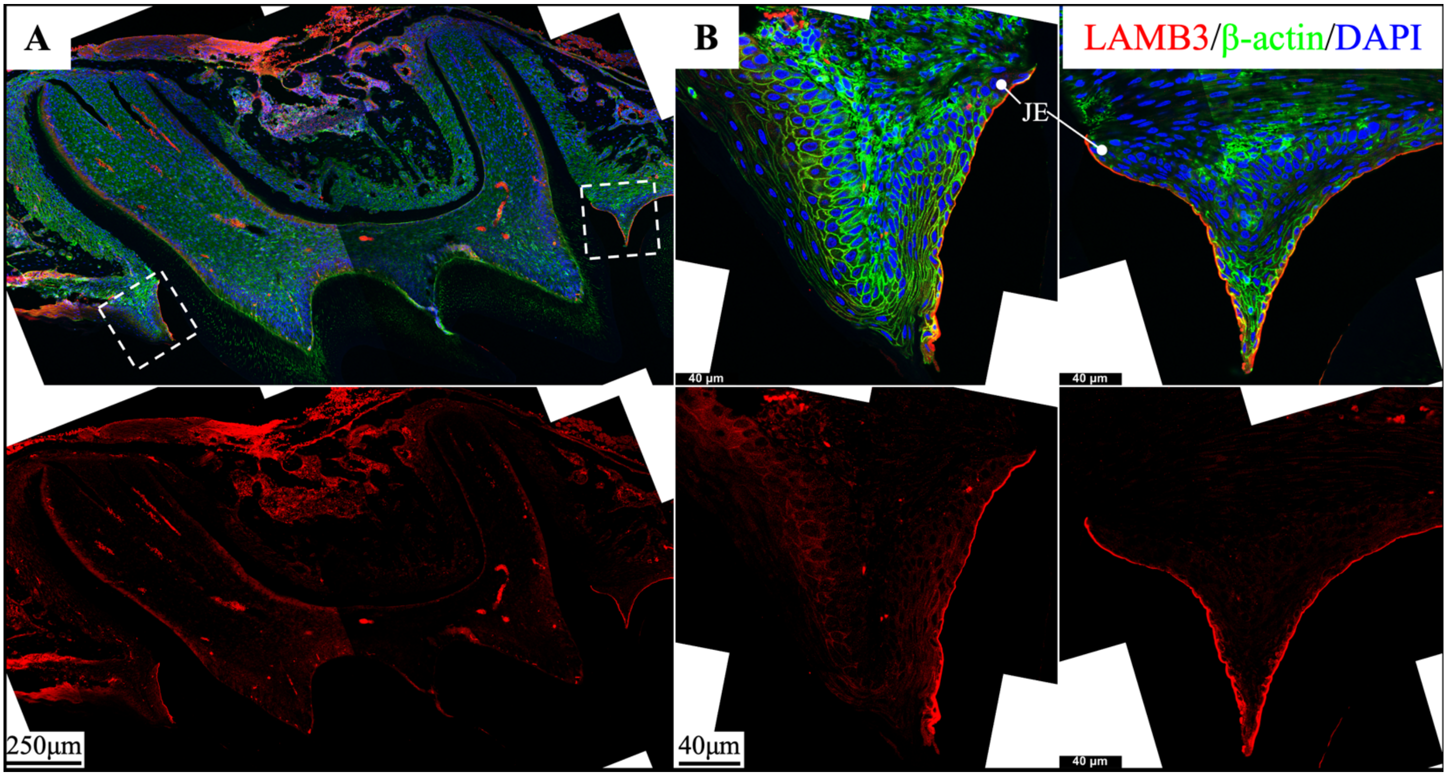

Fig S35. Immunohistochemistry of LAMB3 in 21-day-old mouse maxillary 1st molars. Signal for LAMB3 is red. β-actin signal for cytoskeleton is green. DAPI for nuclei is blue. **A**. The molar has erupted, leaving two sites of junctional epithelium (JE) mesial and distal to the tooth (dashed boxes) on this section. **B**. High magnification images of junctional epithelium.

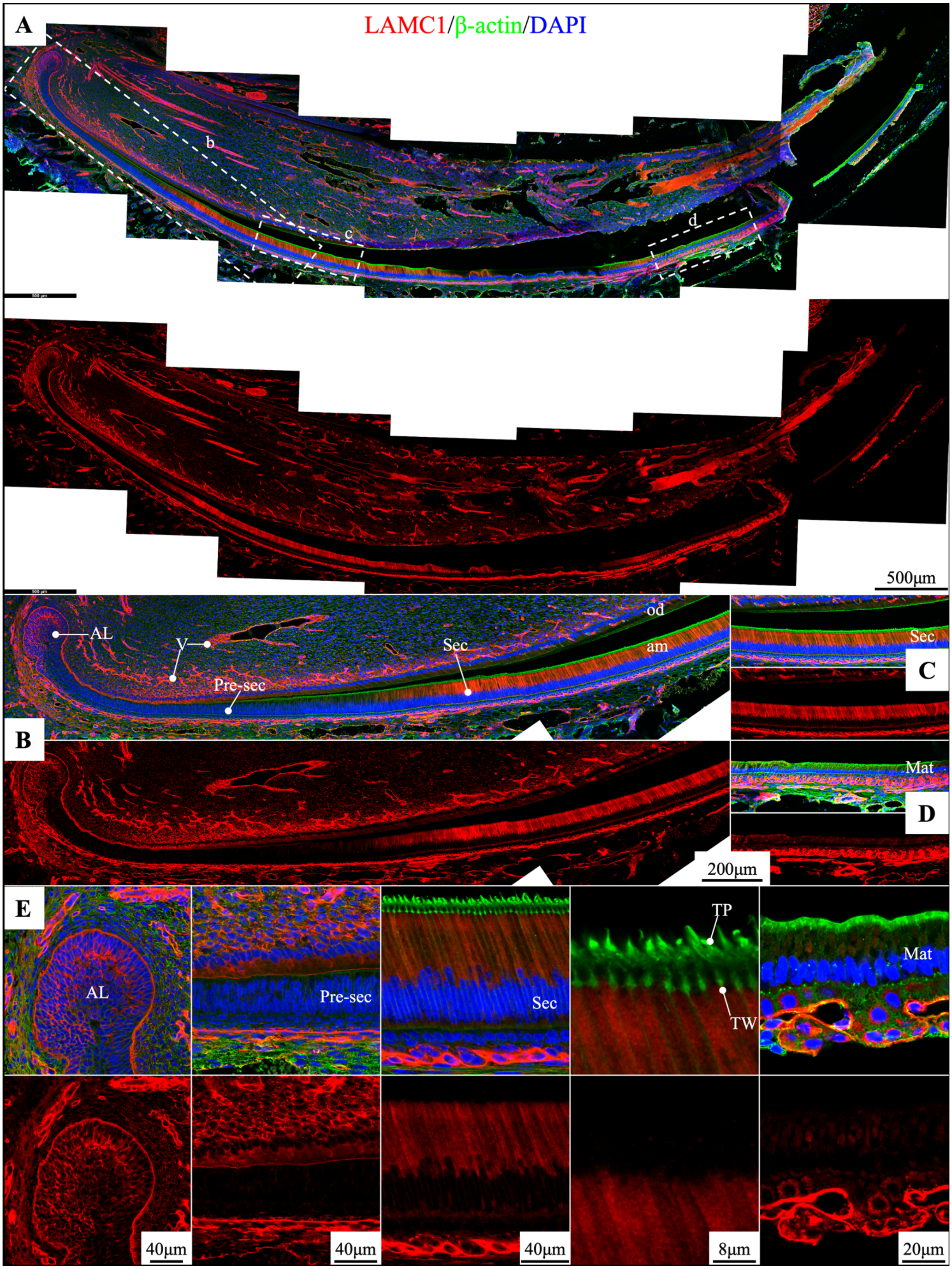

Fig S36. Immunohistochemistry of LAMC1 in 10-day-old mouse mandibular incisors. Signal for LAMC1 is red. β-actin signal for cytoskeleton is green. DAPI for nuclei is blue. Mouse continuously growing incisors contain all developmental stages of ameloblasts (am) (**A**), from the dental epithelial stem cells in the apical loop (AL) to pre-secretory (Pre-Sec), secretory (Sec) (**B&C**), and further into maturation (Mat) stage ameloblasts (**D**). High magnification images are shown in **E**. Key: od, odontoblasts; TP, Tomes' process; TW, (distal) terminal web; V, blood vessels.

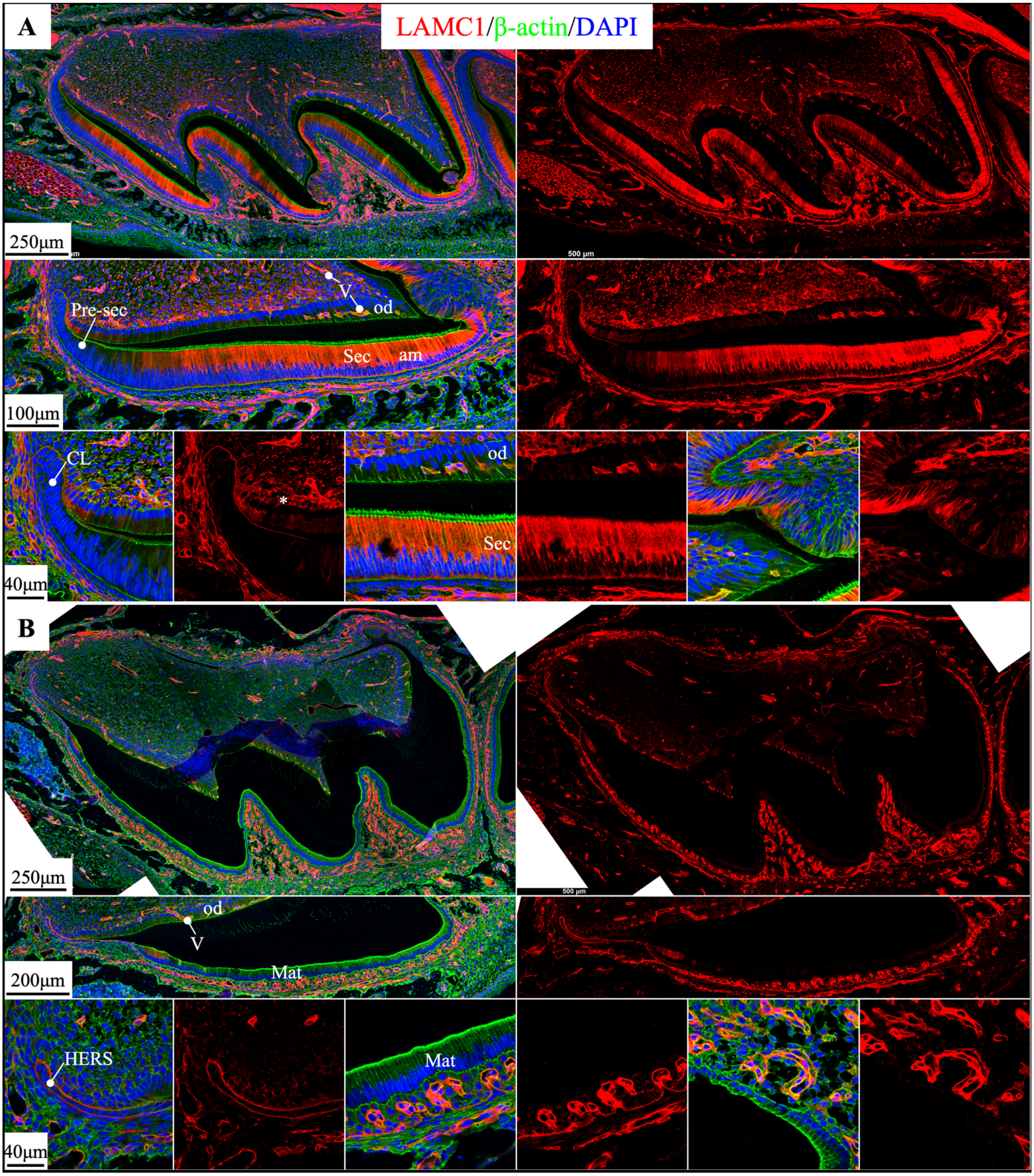

Fig S37. Immunohistochemistry of LAMC1 in 4- (A) and 10-day-old (B) mouse maxillary 1st molars. Signal for LAMC1 is red. β-actin signal for cytoskeleton is green. DAPI for nuclei is blue. **A**. The majority of the 4-day-old enamel organ epithelium is in the secretory (Sec) stage, except for the cervical loop (CL) and pre-secretory (Pre-Sec) stage ameloblasts near the cervical loop. The extracellular matrix of apical dental papilla cells is marked by asterisk (*). **B**. The majority of the 10-day-old enamel organ epithelium is in the maturation (Mat) stage. Hertwig's epithelial root sheath (HERS) is observed at the root aspect of the developing teeth. Key: am, ameloblasts; od, odontoblasts; V, blood vessels.

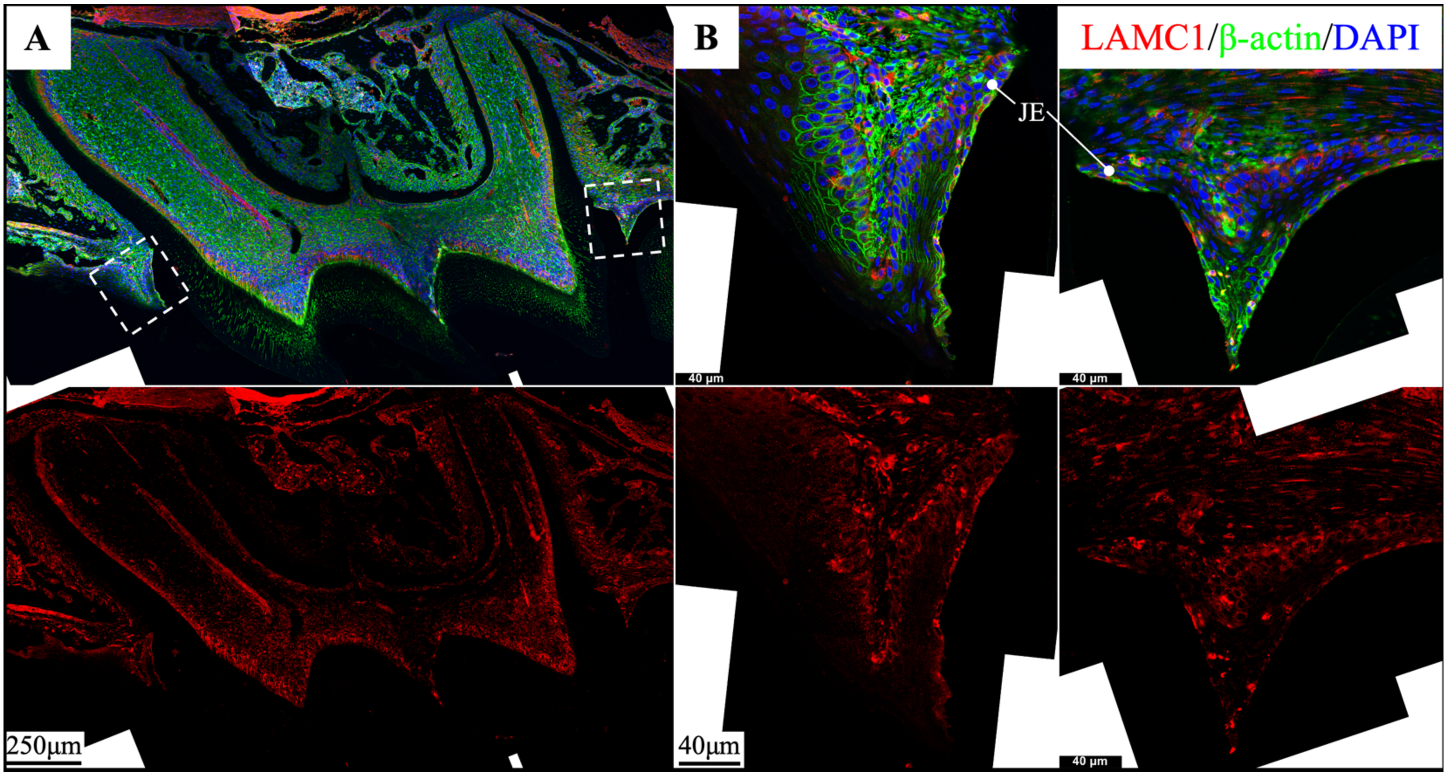

Fig S38. Immunohistochemistry of LAMC1 in 21-day-old mouse maxillary 1st molars. Signal for LAMC1 is red. β-actin signal for cytoskeleton is green. DAPI for nuclei is blue. **A**. The molar has erupted, leaving two sites of junctional epithelium (JE) mesial and distal to the tooth (dashed boxes) on this section. **B**. High magnification images of junctional epithelium.

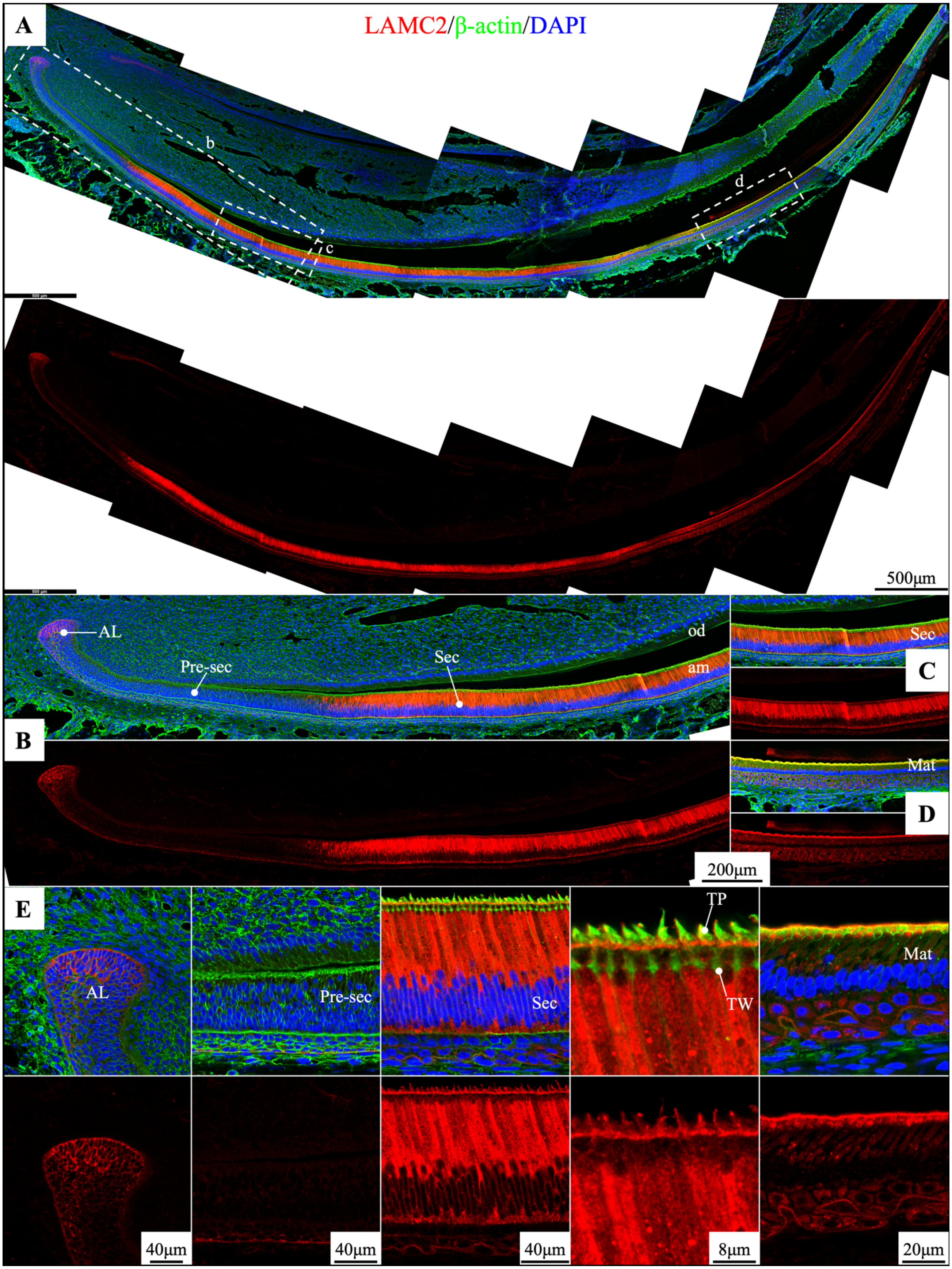

Fig S39. Immunohistochemistry of LAMC2 in 10-day-old mouse mandibular incisors. Signal for LAMC2 is red. β-actin signal for cytoskeleton is green. DAPI for nuclei is blue. Mouse continuously growing incisors contain all developmental stages of ameloblasts (am) (**A**), from the dental epithelial stem cells in the apical loop (AL) to pre-secretory (Pre-Sec), secretory (Sec) (**B&C**), and further into maturation (Mat) stage ameloblasts (**D**). High magnification images are shown in **E**. Key: od, odontoblasts; TP, Tomes' process; TW, (distal) terminal web.

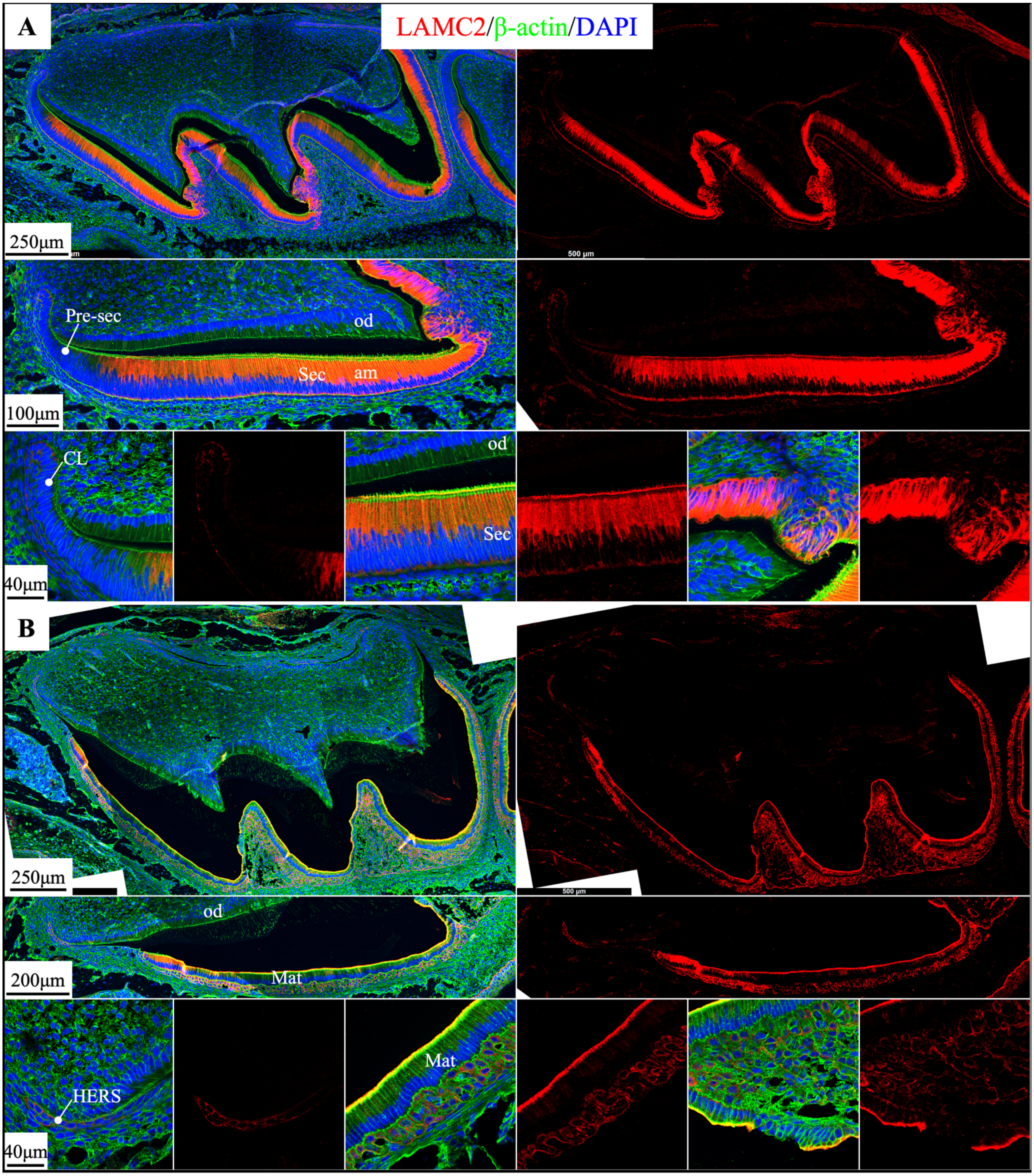

Fig S40. Immunohistochemistry of LAMC2 in 4- (A) and 10-day-old (B) mouse maxillary 1st molars. Signal for LAMC2 is red. β-actin signal for cytoskeleton is green. DAPI for nuclei is blue. **A**. The majority of the 4-day-old enamel organ epithelium is in the secretory (Sec) stage, except for the cervical loop (CL) and pre-secretory (Pre-Sec) stage ameloblasts near the cervical loop. **B**. The majority of the 10-day-old enamel organ epithelium is in the maturation (Mat) stage. Hertwig's epithelial root sheath (HERS) is observed at the root aspect of the developing teeth. Key: am, ameloblasts; od, odontoblasts.

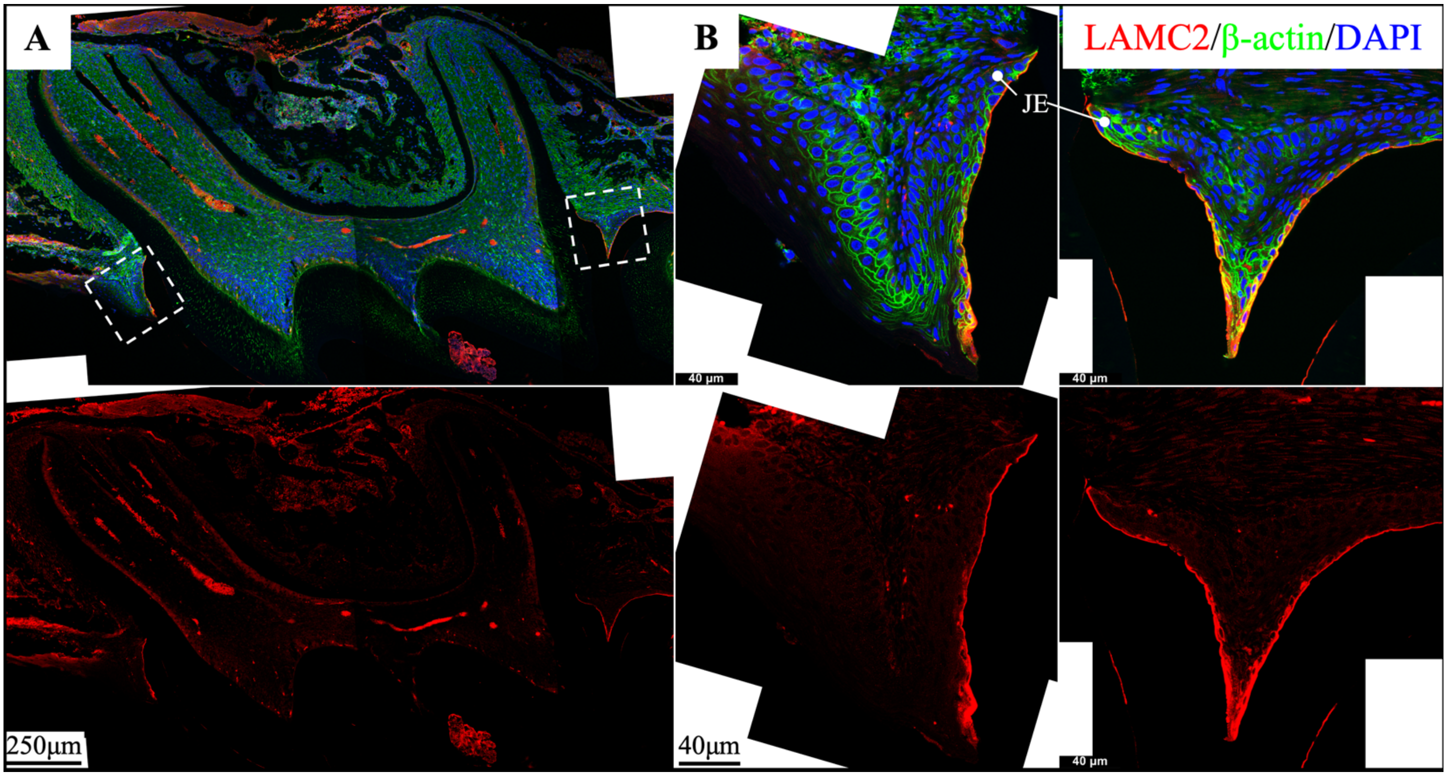

Fig S41. Immunohistochemistry of LAMC2 in 21-day-old mouse maxillary 1st molars. Signal for LAMC2 is red. β-actin signal for cytoskeleton is green. DAPI for nuclei is blue. **A**. The molar has erupted, leaving two sites of junctional epithelium (JE) mesial and distal to the tooth (dashed boxes) on this section. **B**. High magnification images of junctional epithelium.

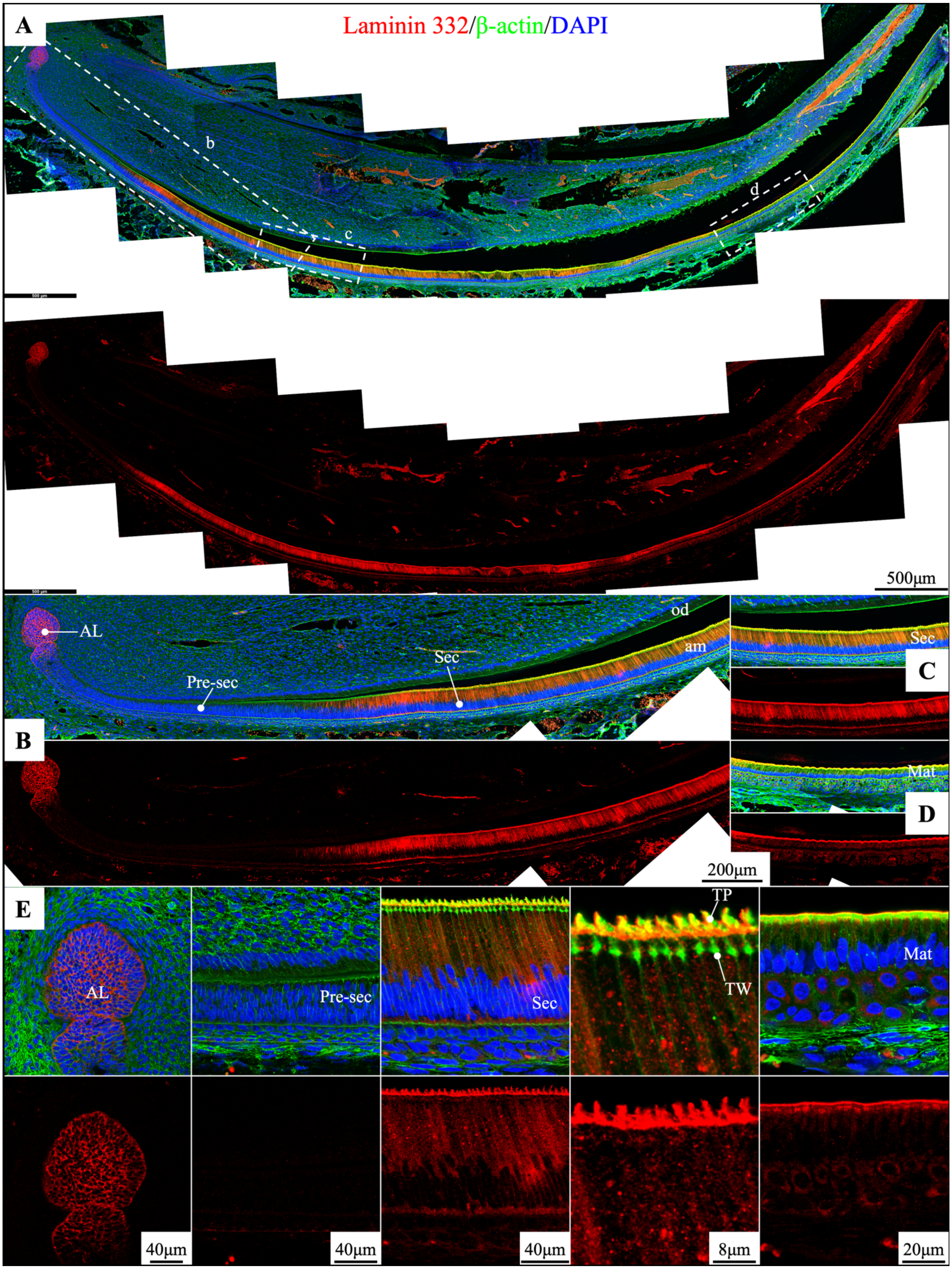

Fig S42. Immunohistochemistry of Laminin 332 in 10-day-old mouse mandibular incisors. Signal for Laminin 332 is red. β-actin signals for cytoskeleton is green. DAPI for nuclei is blue. Mouse continuously growing incisors contain all developmental stages of ameloblasts (am) (**A**), from the dental epithelial stem cells in the apical loop (AL) to pre-secretory (Pre-Sec), secretory (Sec) (**B&C**), and further into maturation (Mat) stage ameloblasts (**D**). High magnification images are shown in **E**. Key: od, odontoblasts; TP, Tomes' process; TW, (distal) terminal web.

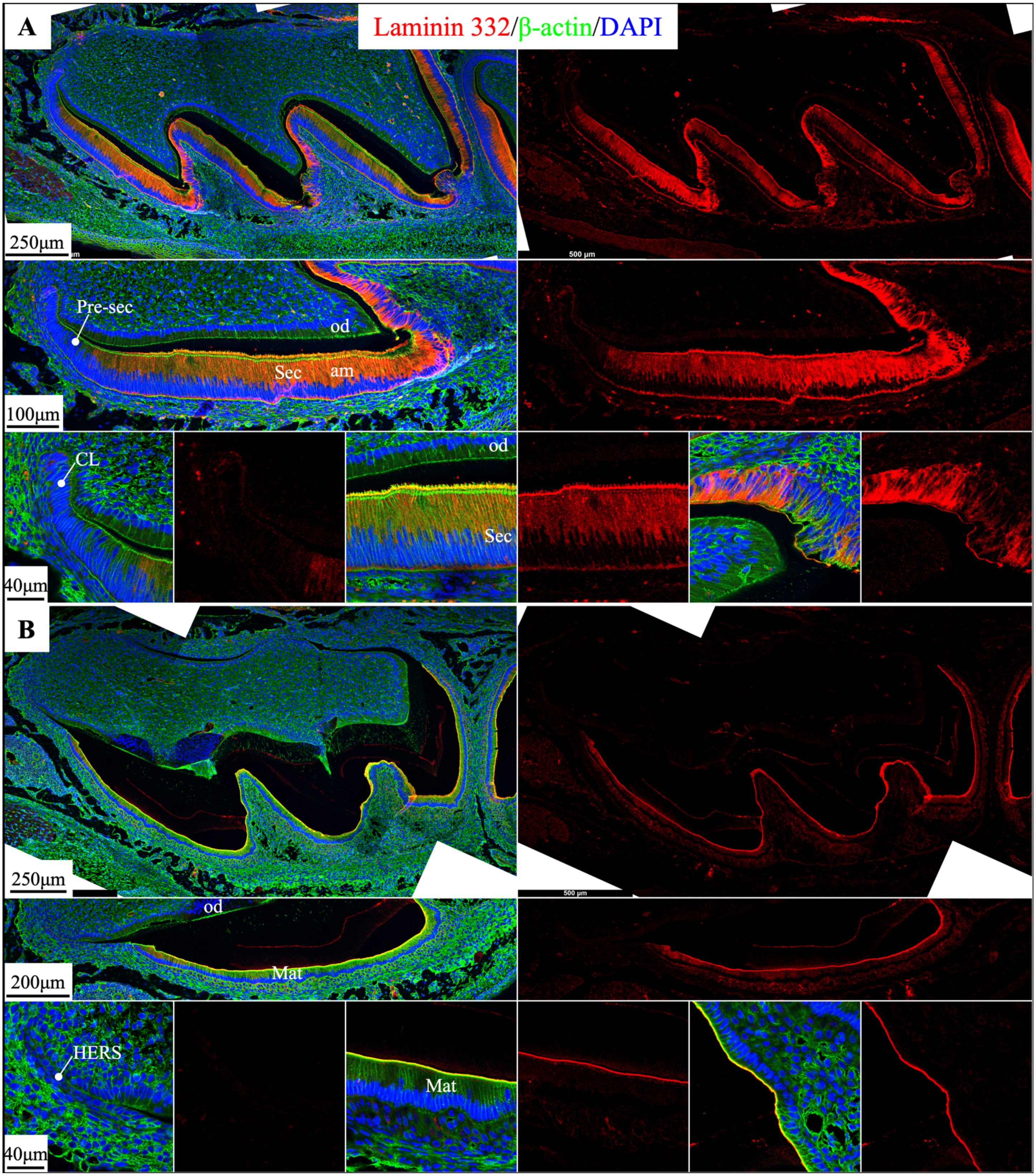

Fig S43. Immunohistochemistry of Laminin 332 in 4- (A) and 10-day-old (B) mouse maxillary 1st molars. Signal for Laminin 332 is red. β-actin signal for cytoskeleton is green. DAPI for nuclei is in blue. **A**. The majority of the 4-day-old enamel organ epithelium is in the secretory (Sec) stage, except for the cervical loop (CL) and pre-secretory ameloblasts (Pre-Sec) near the cervical loop. **B**. The majority of the 10-day-old enamel organ epithelium is in the maturation (Mat) stage. Hertwig's epithelial root sheath (HERS) is observed at the root aspect of the developing teeth. Key: am, ameloblasts; od, odontoblasts.

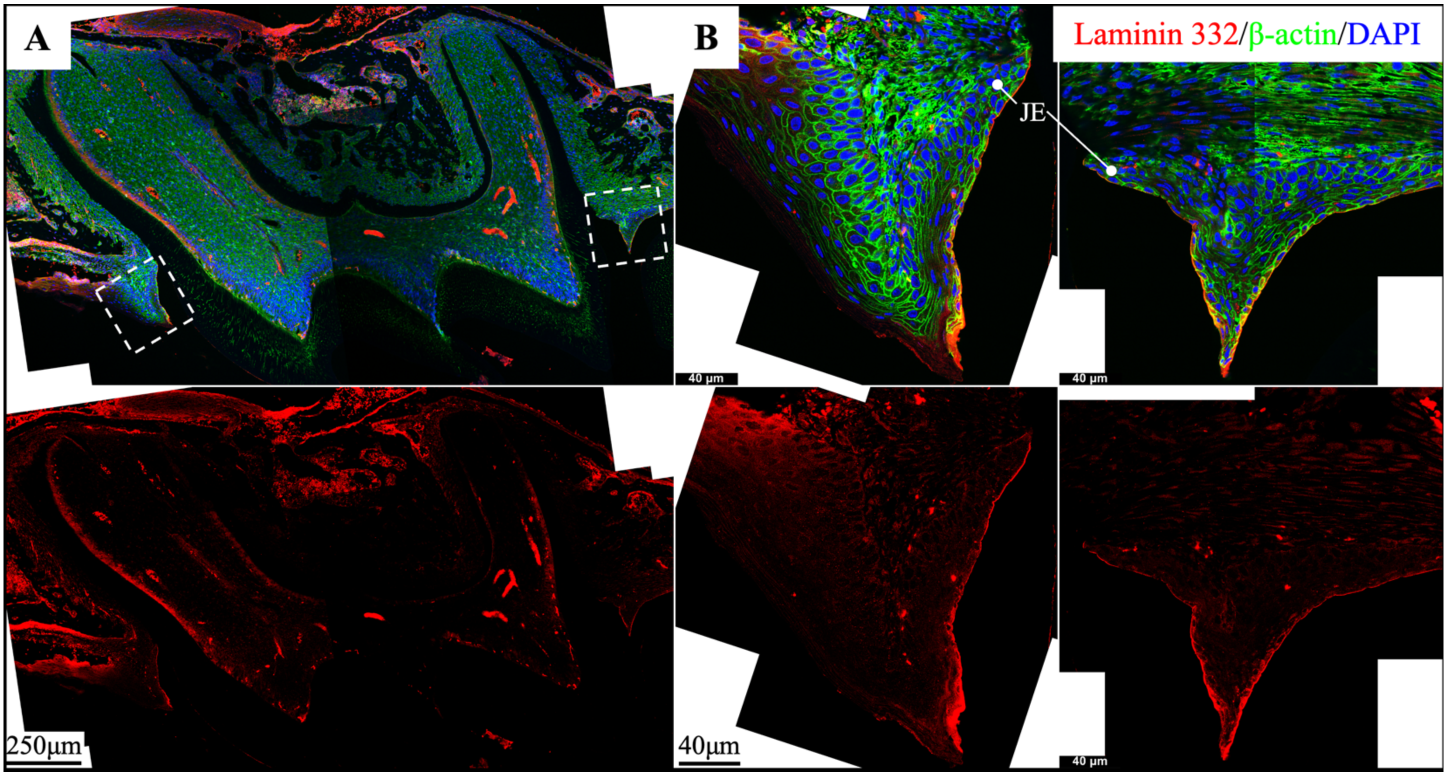

Fig S44. Immunohistochemistry of Laminin 332 in 21-day-old mouse maxillary 1st molars. Signal for Laminin 332 is red. β-actin signal for cytoskeleton is green. DAPI for nuclei is blue. **A**. The molar has erupted, leaving two sites of junctional epithelium (JE) mesial and distal to the tooth (dashed boxes) on this section. **B**. High magnification images of junctional epithelium.
